# N822K- or V560G-mutated KIT activation preferentially occurs in lipid rafts of the Golgi apparatus in leukemia cells

**DOI:** 10.1186/s12964-019-0426-3

**Published:** 2019-09-04

**Authors:** Yuuki Obata, Yasushi Hara, Isamu Shiina, Takatsugu Murata, Yasutaka Tasaki, Kyohei Suzuki, Keiichi Ito, Shou Tsugawa, Kouhei Yamawaki, Tsuyoshi Takahashi, Koji Okamoto, Toshirou Nishida, Ryo Abe

**Affiliations:** 1 Division of Immunobiology, Research Institute for Biomedical Sciences, Tokyo University of Science, Yamazaki 2669, Noda, Chiba 278-0022 Japan; 2 Division of Cancer Differentiation, National Cancer Center Research Institute, Tsukiji 5-1-1, Chuo-ku, 104-0045 Tokyo Japan; 3 Department of Applied Chemistry, Faculty of Science, Tokyo University of Science, Kagurazaka 1-3, Shinjuku-ku, 162-8601 Tokyo Japan; 4 Department of Surgery, Osaka University, Graduate School of Medicine, Yamadaoka 2-2, Suita, Osaka 565-0871 Japan; 5 National Cancer Center Hospital, Tsukiji 5-1-1, Chuo-ku, 104-0045 Tokyo Japan; 6 SIRC, Teikyo University, Itabashi-ku 2-11-1, Itabashi-ku, 173-8605 Tokyo Japan

**Keywords:** Leukemia, KIT tyrosine kinase, Golgi, Lipid rafts, AKT, STAT5, ERK, Endocytosis

## Abstract

**Background:**

KIT tyrosine kinase is expressed in mast cells, interstitial cells of Cajal, and hematopoietic cells. Permanently active *KIT* mutations lead these host cells to tumorigenesis, and to such diseases as mast cell leukemia (MCL), gastrointestinal stromal tumor (GIST), and acute myeloid leukemia (AML). Recently, we reported that in MCL, KIT with mutations (*D816V*, human; *D814Y*, mouse) traffics to endolysosomes (EL), where it can then initiate oncogenic signaling. On the other hand, KIT mutants including KIT^D814Y^ in GIST accumulate on the Golgi, and from there, activate downstream. *KIT* mutations, such as *N822K*, have been found in 30% of core binding factor-AML (CBF-AML) patients. However, how the mutants are tyrosine-phosphorylated and where they activate downstream molecules remain unknown. Moreover, it is unclear whether a KIT mutant other than KIT^D816V^ in MCL is able to signal on EL.

**Methods:**

We used leukemia cell lines, such as Kasumi-1 (*KIT*^*N822K*^, AML), SKNO-1 (*KIT*^*N822K*^, AML), and HMC-1.1 (*KIT*^*V560G*^, MCL), to explore how KIT transduces signals in these cells and to examine the signal platform for the mutants using immunofluorescence microscopy and inhibition of intracellular trafficking.

**Results:**

In AML cell lines, KIT^N822K^ aberrantly localizes to EL. After biosynthesis, KIT traffics to the cell surface via the Golgi and immediately migrates to EL through endocytosis in a manner dependent on its kinase activity. However, results of phosphorylation imaging show that KIT is preferentially activated on the Golgi. Indeed, blockade of KIT^N822K^ migration to the Golgi with BFA/M-COPA inhibits the activation of KIT downstream molecules, such as AKT, ERK, and STAT5, indicating that KIT signaling occurs on the Golgi. Moreover, lipid rafts in the Golgi play a role in KIT signaling. Interestingly, KIT^V560G^ in HMC-1.1 migrates and activates downstream in a similar manner to KIT^N822K^ in Kasumi-1.

**Conclusions:**

In AML, KIT^N822K^ mislocalizes to EL. Our findings, however, suggest that the mutant transduces phosphorylation signals on lipid rafts of the Golgi in leukemia cells. Unexpectedly, the KIT^V560G^ signal platform in MCL is similar to that of KIT^N822K^ in AML. These observations provide new insights into the pathogenic role of KIT mutants as well as that of other mutant molecules.

**Electronic supplementary material:**

The online version of this article (10.1186/s12964-019-0426-3) contains supplementary material, which is available to authorized users.

## Background

KIT is a member of the type III receptor tyrosine kinase (RTK) family that includes platelet-derived growth factor receptor A/B (PDGFRA/B), fms, and fms-like tyrosine kinase 3 (FLT3) [1–3]. It is known to participate in tyrosine phosphorylation signals at the PM, ensuring cell growth and survival in hematopoietic cells, mast cells, interstitial cells of Cajal, germ cells, and melanocytes [4–6].

KIT is composed of *N*-glycosylated immunoglobulin domains in the amino-terminal extracellular portion, transmembrane region, juxta-membrane (JM) region, and the carboxy-terminal cytoplasmic tyrosine kinase domain [6, 7]. Stem cell factor (SCF), a ligand for KIT, stimulates KIT phosphorylation on selective tyrosine residues, such as Y703 and Y721, and these phospho-tyrosines serve as docking sites for downstream molecules [7–9]. SCF-KIT activates the phosphatidyl 3-kinase-AKT pathway and the RAS-MEK-ERK cascade, which control gene expression, metabolism, and cytoskeletal architecture [6, 9–11]. The JM region plays a role in autoinhibition of the receptor through *intra*-molecular binding [12]. Thus, constitutively active mutations of *KIT* allow host cells to autonomously proliferate, resulting in the development of AML, MCL, GIST, germ cell tumors, and melanoma [6, 13–16]. In particular, *KIT* mutations in the JM region (eg, *V560G*, *deletion* etc.) are found in 70% of GIST patients [17–19]. A tyrosine kinase inhibitor, imatinib mesylate (Gleevec), has been developed for the treatment of GIST, and it has dramatically improved the prognosis of patients [19, 20]. However, *KIT*-bearing mutations in the kinase activation loop (AL), such as *D816V*, cause a loss of sensitivity to imatinib [17, 18, 21, 22]. In comparison to JM mutants (mut), KIT^N822K^ is also imatinib-resistant but to a lesser degree than KIT^D816V^ [17, 22, 23].

Previously, we reported that in MCL, KIT^D816V^ (human) and KIT^D814Y^ (mouse) activate AKT and the signal transducer and activator of transcription 5 (STAT5) in EL and on the ER, respectively (Table 1) [24, 25]. Furthermore, previous studies showed that in cells other than those of MCL, such as GIST and NIH3T3 cells, JM-mut or AL-mut accumulates on the Golgi apparatus, where it initiates oncogenic signals (Table 1) [26–29]. These studies raised important questions as to whether mutant KIT initiates signaling from intracellular compartments in other cancers such as AML, and whether the mutation status of *KIT* affects the platform for oncogenic signaling.

**Table 1:** Summary of KIT localization and signaling in AML, MCL, and GIST

Cell line, KIT mutation	KIT localization	Downstream activation	Reference
Kasumi-1 (AML), *N822K*	EL	Golgi	This study
HMC-1.1 (MCL), *V560G*	EL	Golgi	
HMC-1.2/RCM (MCL), *D816V*/*D814Y*	EL	ER, EL	[24, 25]
GIST cell lines (*ex11*/*17* etc…), NIH3T3 (transfected *KIT*^*D816V*^ etc…)	Golgi	Golgi	[26–29, 30]

*AML* acute myeloid leukemia, *MCL* mast cell leukemia, *GIST* gastrointestinal stromal tumor, *Ex* exon, *EL* endo-lysosomes, *ER* endoplasmic reticulum

*KIT* mutations have been found in approximately 30% of CBF-AML patients who have chromosome aberrations [31–33]. Recent studies showed that active *KIT* mutations are correlated with a poor prognosis in AML patients [31, 32]. The major activating *KIT* mutations are found at D816 and N822 (26 cases and 14 cases in 63 *KIT* mutation-positive patients, respectively) [33]. Although spatio-temporal analyses of KIT^D816V^ signals have been performed [24, 25, 28], it is unclear whether the *N822K* mutation in leukemia affects KIT localization and the signal platform.

We then investigated the relationship between KIT^N822K^ localization and tyrosine phosphorylation signals in Kasumi-1 cells (an AML cell line) that endogenously express KIT^N822K^. Furthermore, we examined whether KIT^V560G^ in HMC-1.1 (MCL) caused signaling on the Golgi, ER, PM, or EL. In Kasumi-1, KIT is preferentially found in EL. Newly synthesized KIT in the ER traffics to the PM through the Golgi and subsequently relocates to EL through endocytosis in a manner dependent on its kinase activity. Our immunofluorescence assay, however, showed that KIT autophosphorylation preferentially occurs on the Golgi. Indeed, KIT^N822K^ activates AKT, ERK, and STAT5 on the Golgi in Kasumi-1 cells. Moreover, lipid rafts in the Golgi play a role in KIT signaling. Interestingly, KIT^V560G^ in MCL transduces signals in the Golgi in a similar manner to KIT^N822K^ in AML but not to KIT^D816V^ in MCL. Our study demonstrates that both KIT^N822K^ and KIT^V560G^ are mainly present in EL, but that their signal platform in leukemia cells is the lipid rafts of the Golgi. Furthermore, blockade of mutant KIT incorporation into the lipid rafts may provide a new strategy for suppression of growth signals in leukemia cells.

## Methods

### Cell culture

Kasumi-1, SKNO-1 (JCRB Cell Bank, Osaka, Japan), HMC-1.1 (Merck Millipore, Darmstadt, Germany), HMC-1.2 and pt18 cells were cultured at 37 °C in RPMI1640 medium supplemented with 10% FCS, penicillin, streptomycin, glutamine (Pen/Strep/Gln), and reducing agents (0.5 mM monothioglycerol or 50 μM 2-mercaptoethanol). For expansion of SKNO-1, 10 ng/mL granulocyte macrophage colony-stimulating factor (GM-CSF, Peprotech, Rocky Hill, NJ) was used. GIST-T1 cells (Cosmo Bio, Tokyo, Japan) were cultured at 37 °C in DMEM supplemented with 10% FCS and Pen/Strep/Gln. All human cell lines were authenticated by Short Tandem Repeat analysis at JCRB Cell Bank (Osaka, Japan) and tested for *Mycoplasma* contamination with a MycoAlert Mycoplasma Detection Kit (Lonza, Basel, Switzerland).

### Chemicals

Imatinib (Cayman Chemical, Ann Arbor, MI) and PKC412 (Selleck, Houston, TX) were dissolved in DMSO. Bafilomycin A1, brefeldin A (Sigma, St. Louis, MO), monensin (Biomol, Exeter, UK), and cer-C6 (Cayman Chemical) were dissolved in ethanol. M-COPA (also known as AMF-26) was synthesized as previously described [34, 35] and dissolved in DMSO.

### Antibodies

The sources of purchased antibodies were as follows: KIT (M^− 14^), cathepsin D (H^− 75^), STAT5 (C-17), ERK2 (K-23), ARF1 (ARFS 3F1), GBF1 (25), PTP1B (D-4), SHP-1 (D-11), and SHP-2 (B-1) from Santa Cruz Biotechnology (Dallas, TX); KIT [pY703] (D12E12), KIT (D13A2), LAMP1 (lysosome-associated membrane protein 1, D4O1S), AKT (40D4), AKT [pT308] (C31E5E), STAT5 (D2O6Y), STAT5[pY694] (D47E7), ERK1/2 (137F5) and ERK [pT202/pY204] (E10) from Cell Signaling Technology (Danvers, MA); PDI (RL90), TFR (transferrin receptor, ab84036), giantin (ab24586), and GM130 (EP892Y) from Abcam (Cambridge, UK); TFR (H68.4) from Thermo Fisher Scientific (Rockford, IL); calnexin (ADI-SPA-860) from Enzo (Farmingdale, NY); GM130 (clone 35) from BD Transduction Laboratories (Franklin Lakes, NJ); LAMP1 (L1418) from Sigma (St. Louis, MO) and KIT (104D2) from Biolegend (San Diego, CA). HRP-labeled secondary antibodies were purchased from the Jackson Laboratory (Bar Harbor, MA). Alexa Fluor-conjugated secondary antibodies were obtained from Molecular Probes (Eugene, OR).

### Immunofluorescence confocal microscopy

Cells in suspension culture were fixed with methanol for 10 min at − 20 °C or with 4% paraformaldehyde for 20 min at room temperature, then cyto-centrifuged onto coverslips. GIST-T1 cells were cultured on poly-L-lysine-coated coverslips and fixed as above. Fixed cells were permeabilized and blocked for 30 min in PBS supplemented with 0.1% saponin and 3% BSA, and then incubated with a primary and a secondary antibody for 1 h each. After washing with PBS, cells were mounted with Fluoromount (DiagnosticBioSystems, Pleasanton, CA). Confocal images were obtained with an Fluoview FV10i (Olympus, Tokyo, Japan) or a TCS SP5 II/SP8 (Leica, Wetzlar, Germany) laser scanning microscope. Composite figures were prepared with Photoshop Elements 10 and Illustrator CS6 software (Adobe, San Jose, CA). Pearson’s R were calculated with NIH ImageJ 1.48v software.

### Western blotting

Lysates prepared in SDS-PAGE sample buffer were subjected to SDS-PAGE and electro-transferred onto PVDF membranes. Immunodetection was performed by ECL (PerkinElmer, Waltham, MA). Sequential re-probing of membranes was performed after the complete removal of primary and secondary antibodies in stripping buffer (Thermo Fisher Scientific), or inactivation of peroxidase by 0.1% sodium azide. Results were analyzed with an LAS-3000 with Science Lab software (Fujifilm, Tokyo, Japan) or a ChemiDoc XRC+ with Image Lab software (BIORAD, Hercules, CA).

### Flow cytometry

Leukemia cells were directly collected from a cell suspension by centrifugation. GIST-T1 cells were scraped from culture dishes and then centrifugated. Cells were stained with anti-KIT (104D2) and Alexa Fluor 488-conjugated anti-mouse IgG in PBS supplemented with 0.5% calf serum and 0.1% NaN_3_ at 4 °C for 1 h each. Stained cells were fixed with 4% paraformaldehyde for 20 min at room temperature. Flowcytometric data were obtained by FACSCalibur (BD Biosciences, Franklin Lakes, NJ), and results were analyzed with FlowJo software (Tomy Digital Biology, Tokyo, Japan).

### Gene silencing with small interfering RNA (siRNA)

For silencing *ARF1, GBF1, PTP1B, SHP-1, or SHP-2* genes, ON-TARGETplus SMARTpool siRNAs were purchased from Dharmacon (Lafayette, CO). ON-TARGETplus Non-targeting Pool (Dharmacon) was used as a source of control siRNAs. Electroporation was performed using a Neon Transfection System (Thermo Fisher Scientific), according to the manufacturer’s instructions.

### Cell proliferation assay

To measure Kasumi-1 and HMC-1.1 proliferation, cells were cultured with Gleevec or Midostaurin (referred to henceforth as imatinib and PKC412, respectively) for 18 h, and then treated with [^3^H] thymidine deoxyribonucleotide (TdR) for 12 h. Cell proliferation was evaluated by the incorporation of [^3^H] TdR.

### Analysis of protein glycosylation

Following the manufacturer’s instructions (New England Biolabs, Ipswich, MA), NP-40 cell lysates were treated with endoglycosidases for 1 h at 37 °C. The reactions were stopped with SDS-PAGE sample buffer, and products were resolved by SDS-PAGE and immunoblotted.

### Statistical analyses

For statistical analysis, experiments were repeated as three biological replicates. Differences between two or more groups were analyzed by the two-tailed Student’s *t*-test or one-way analysis of variance followed by Dunnett’s multiple comparison *post-hoc* test, respectively. All significant differences stated indicate a 5% level of probability.

## Results

### KIT^N822K^ and KIT^V560G^ mislocalize to EL in leukemia cells

**Fig. 1:**
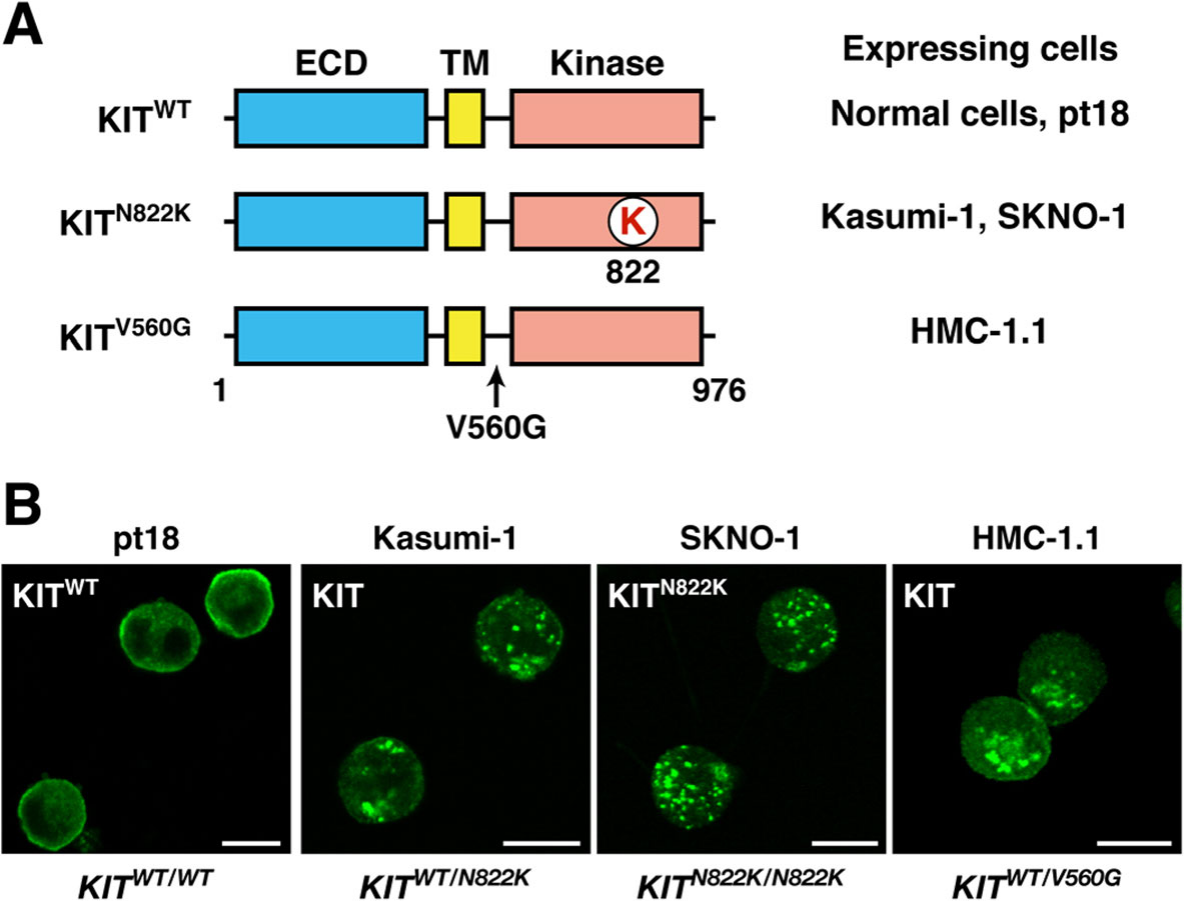
N822K- or V560G-mutated KIT mis-localizes to vesicular structures in leukemia cells. **a** Schematic representations of wild-type KIT (KIT^WT^) and constitutively active KIT mutants (KIT^N822K^ and KIT^V560G^) showing the extracellular domain (ECD), the transmembrane domain (TM), the kinase domain, the lysine mutation at 822 (K in red), and the glycine mutation at 560 (V560G). **b** Kasumi-1, SKNO-1, HMC-1.1, or pt18 cells were immunostained with anti-KIT. Bars, 10 μm. Note that KIT^WT^ localized to the PM, whereas KIT mutants accumulated on vesicular compartments

**Fig. 2:**
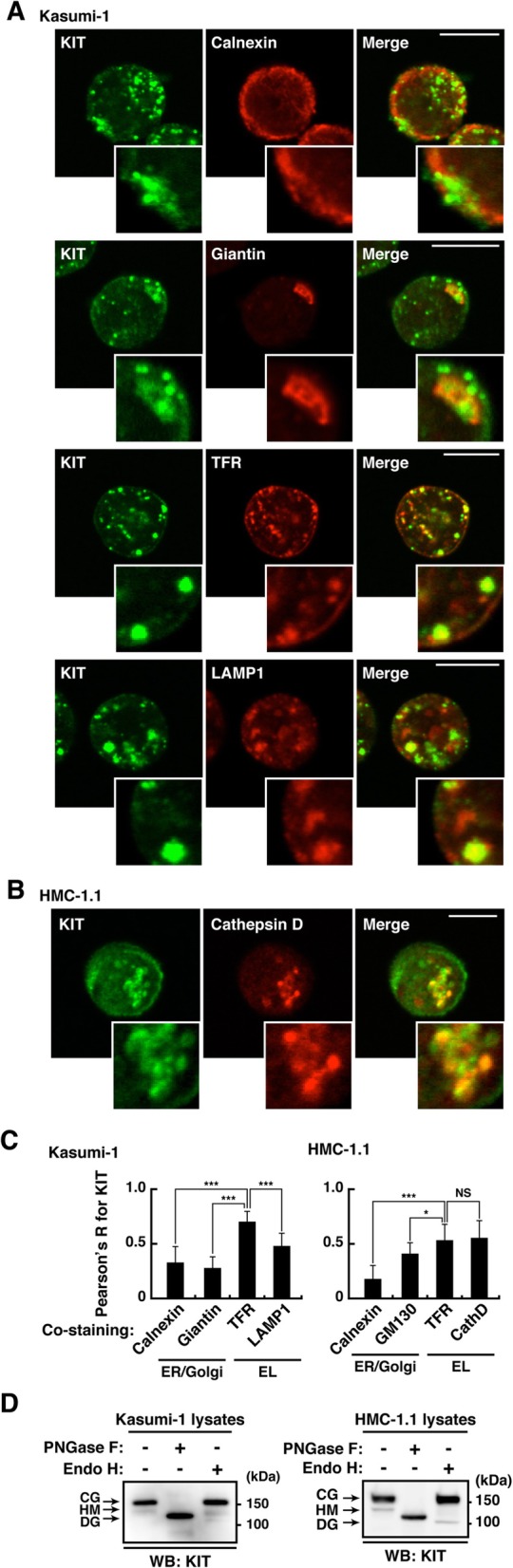
KIT mutants localize to EL but not to the PM in leukemia cells. **a** & **b** Kasumi-1 (**a**) or HMC-1.1 cells (**b**) were double-stained with anti-KIT (green) plus the indicated antibody (red). Insets show magnified images. Bars, 10 μm. **c** Pearson’s R correlation coefficients were calculated by analyzing the intensity of KIT vs. organelle markers. Results are means ± s.d. (*n* = 12~22). *P < 0.05, ***P < 0.001. NS, not significant. Calnexin (ER marker); giantin (Golgi marker); GM130 (Golgi marker); TFR (endosome marker); LAMP1 (lysosome marker); cathepsin D (cathD, lysosome marker). **d** Lysates from Kasumi-1 (left) or HMC-1.1 cells (right) were treated with peptide N-glycosidase F (PNGase F) or endoglycosidase H (endo H) then immunoblotted with anti-KIT. CG, complex-glycosylated form; HM, high mannose form; DG, deglycosylated form. Note that most KIT was present in a complex-glycosylated form in these leukemia cells

To investigate the sub-cellular localization of endogenous KIT, we performed confocal immunofluorescence microscopic analyses in pt18 (mouse mast cell line, *KIT wild-type (WT)*), Kasumi-1 (human AML, *KIT*^*WT/N822K*^), SKNO-1 (human AML, *KIT*^*N822K/N822K*^), and HMC-1.1 (human MCL, *KIT*^*WT/V560G*^) (Fig. 1a). As previously described [24], most KIT^WT^ localized to the PM in pt18 (Fig. 1b, left). In contrast, KIT accumulated in vesicular structures in Kasumi-1, SKNO-1, and HMC-1.1 (Fig. 1b). We then performed co-staining assays to identify these structures. In Kasumi-1, KIT was co-localized with TFR (transferrin receptor, endosome marker) and LAMP1 (lysosome marker) rather than with calnexin (ER marker) or giantin (Golgi marker) (Fig. 2a). Similarly, in HMC-1.1, KIT in vesicular structures was colocalized with TFR and cathepsin D (lysosome marker) (Fig. 2b; Additional file 1: Figure S1A). By calculating Pearson’s R correlation coefficients (Pearson’s R) for KIT versus organelle markers, the intensity from KIT-TFR was significantly greater than that from KIT-calnexin, −giantin, and -LAMP1 in Kasumi-1 (Fig. 2c, left graph), suggesting that KIT mainly localizes to endosomes. The quantification showed that in HMC-1.1, KIT was colocalized with TFR to a similar extent as with cathepsin D (Fig. 2c*,* right graph). In both types of cells, KIT was colocalized with EL markers rather than with ER/Golgi markers. We previously showed that in MCL and GISTs, KIT mutants are normally complex-glycosylated in the Golgi [24, 26]. To test for the KIT glycosylation state in Kasumi-1 and HMC-1.1, we treated KIT with endoglycosidase H, which digests immature high-mannose forms of KIT but not mature complex-glycosylated forms. Figure 2d shows that most KIT in these leukemia cells was in a complex-glycosylated form, similar to normal KIT [24, 28, 29]. KIT shifted to a non-glycosylated form following the complete digestion of N-linked glycans by peptide-N-glycosidase F. In SKNO-1, as with Kasumi-1 and HMC-1.1, KIT was complex-glycosylated (Additional file 1: Figure S1B). Pearson’s R quantification from immunofluorescence images showed that in SKNO-1, KIT localized to endosomes to a similar extent as to lysosomes and it was found in EL rather than on ER/Golgi (Additional file 1: Figure S1C & D). These results suggest that complex-glycosylated KIT accumulates in EL in leukemia cells but not in the early secretory compartments.

### KIT mutants autonomously migrate from the PM to EL through endocytosis in a manner dependent on their kinase activity

**Fig. 3:**
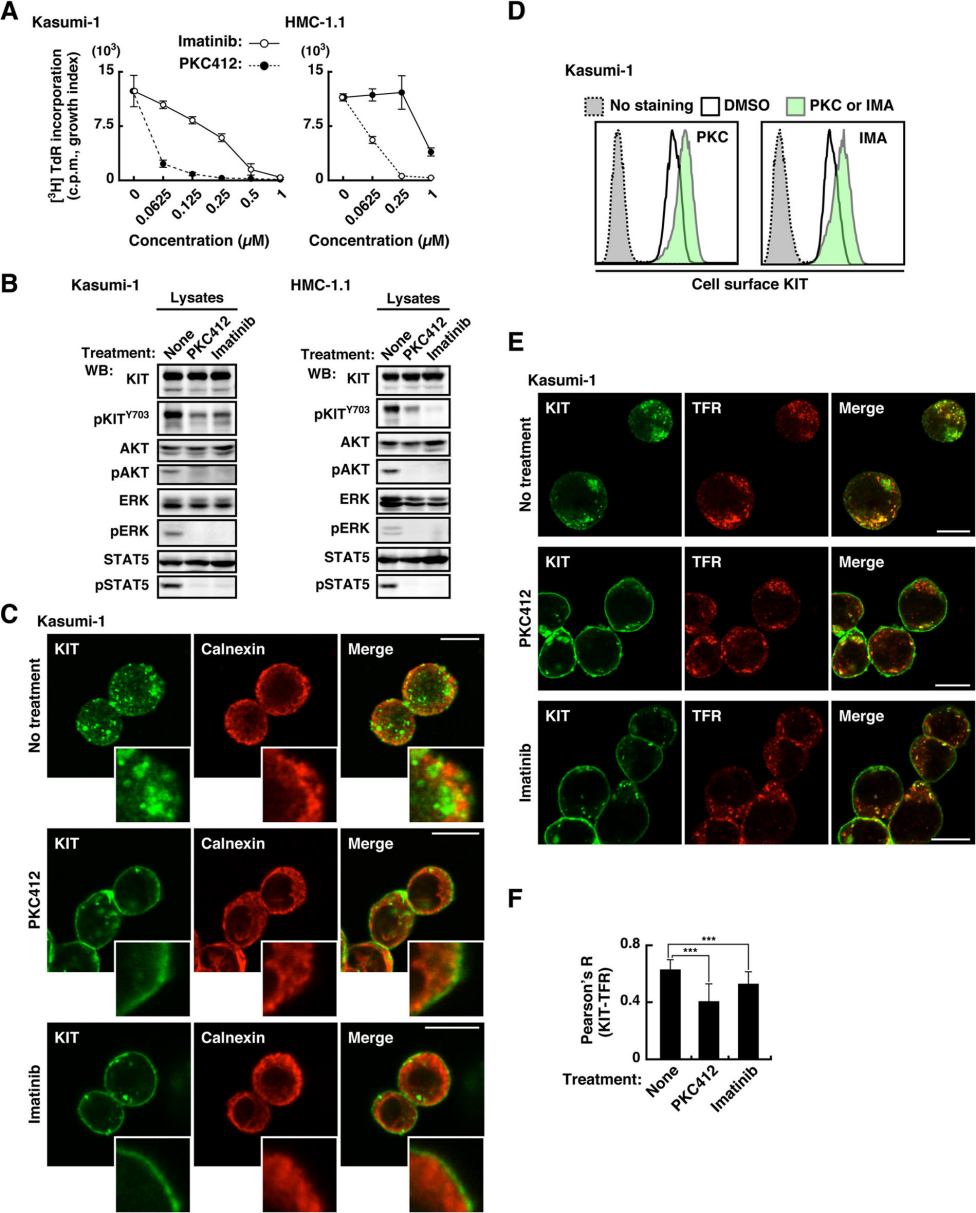
KIT migrates to EL through endocytosis in a manner dependent on their kinase activity. **a** Kasumi-1 or HMC-1.1 cells were cultured for 24 h in the presence of KIT kinase inhibitors (imatinib, open circles; PKC412, closed circles). The graphs show the levels of [^3^H] thymidine deoxyribonucleotide (TdR) incorporation into cells (counts per minute, c.p.m., growth index) at the indicated inhibitor concentrations. Results are means ± s.d. (*n* = 3). **b** Kasumi-1 or HMC-1.1 cells were treated for 4 h with 1 μM PKC412 or 1 μM imatinib. Lysates were immunoblotted for KIT, phospho-KIT Y703 (pKIT^Y703^), AKT, pAKT, STAT5, pSTAT5, ERK, and pERK. **c**-**f** Kasumi-1 cells were treated for 12 h with 1 μM PKC412 (PKC) or 1 μM imatinib (IMA). **c** Cells were immunostained with anti-KIT (green) and anti-calnexin (ER marker, red). Confocal immunofluorescence images are shown. Insets show magnified images of the PM region. Bars, 10 μm. **d** Cell surface KIT levels determined by flow cytometry are shown. Non-permeabilized cells were stained with anti-KIT extracellular domain antibody. Green histogram, with KIT inhibitor treatment; white histogram, no KIT inhibitor; gray histogram, no anti-KIT antibody control. **e** Cells were immunostained with anti-KIT (green) and anti-TFR (endosome marker, red). Confocal immunofluorescence images are shown. Bars, 10 μm. **f** Pearson’s R correlation coefficients were calculated by analyzing the intensity of KIT vs. TFR. Results are means ± s.d. (*n* = 22~29). ***P < 0.001. Note that these inhibitors lowered KIT in vesicular structures and increased KIT in the PM

Next, we examined the role of KIT kinase activity on cell proliferation, growth signals, and KIT localization. As previously reported [36–39], Kasumi-1 and HMC-1.1 proliferate autonomously, and KIT tyrosine kinase inhibitors (TKIs), such as imatinib and PKC412, suppressed cell proliferation in a dose-dependent manner (Fig. 3a). Immunoblotting showed that phosphorylation of KIT, AKT, ERK, and STAT5 occurred in the absence of TKIs, and PKC412 and imatinib reduced these phosphorylations (Fig. 3b), confirming that KIT activates AKT, ERK, and STAT5 in Kasumi-1 and HMC-1.1. Next, we investigated the localization of KIT in TKI-treated cells by immunofluorescence and flow cytometry. In Kasumi-1 cells treated with TKIs, KIT localized more in PM (outside ER staining, Fig. 3c & d) and less in endosomes (Fig. 3e & f). Similar results were seen with HMC-1.1 and SKNO-1 (Additional file 1: Figure S1E). Collectively, in these leukemia cells, newly synthesized KIT in the ER moves to the PM along the secretory pathway and subsequently traffics to EL through kinase activity-dependent endocytosis. In addition to our previous findings that KIT^D816V^ and KIT^⊿560–578^ in the PM are increased by TKI treatment [24, 26, 40], these results suggest that retention in the PM by TKIs is a ubiquitous feature of KIT mutants.

### Autophosphorylation of KIT^N822K^ and KIT^V560G^ predominantly occurs on the Golgi in leukemia cells

**Fig. 4:**
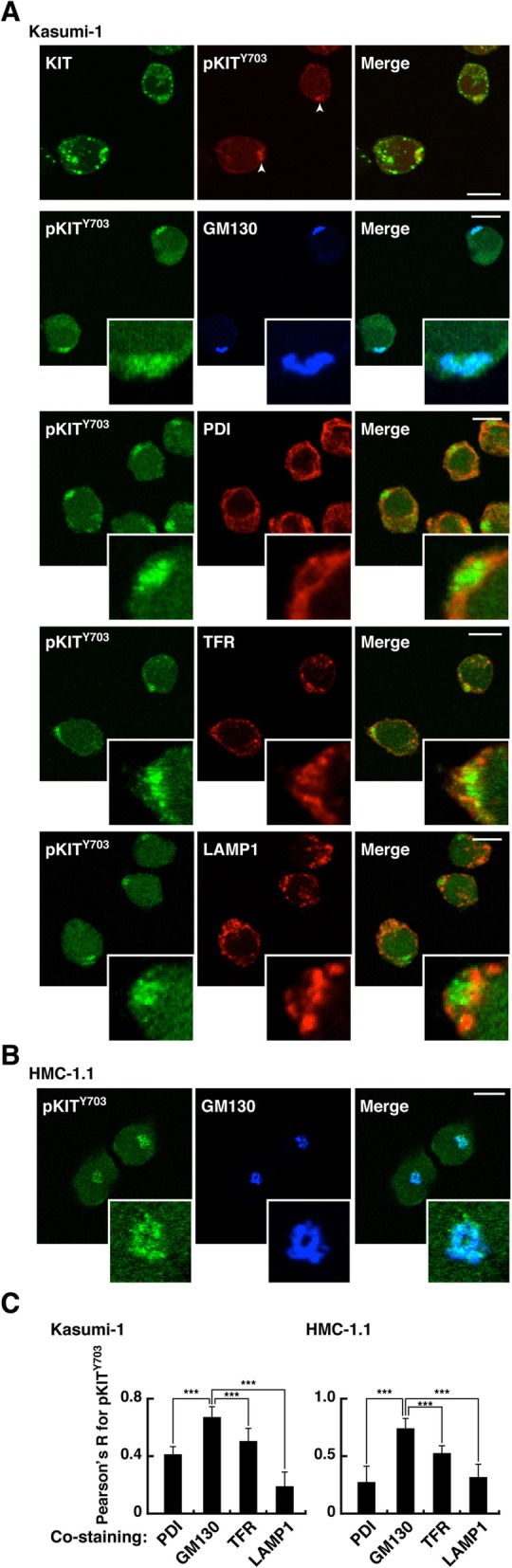
Autophosphorylation of KIT^N822K^ and KIT^V560G^ occurs preferentially on the Golgi in leukemia cells. **a** & **b** Kasumi-1 (**a**) or HMC-1.1 cells (**b**) were immunostained for KIT (green), phospho-KIT Y703 (pKIT^Y703^, red or green) together with GM130 (Golgi marker, blue), PDI (protein disulfide isomerase, ER marker, red), TFR (endosome marker, red), or LAMP1 (lysosome marker, red). Insets show magnified images. Bars, 10 μm. **c** Pearson’s R correlation coefficients were calculated by analyzing the intensity of pKIT^Y703^ vs. organelle markers. Results are means ± s.d. (*n* = 12~21). ***P < 0.001. Note that pKIT^Y703^ was colocalized with GM130 rather than with PDI, TFR, or LAMP1 both in Kasumi-1 and HMC-1.1 cells

We next examined the site of KIT activation in leukemia cells. To determine the signal platform for KIT, we immuno-stained for phospho-tyrosine residues in the kinase domain which would indicate KIT activation [7–9, 26, 27]. In Kasumi-1 cells, phospho-KIT Y703 (pKIT [Y703]) was clearly detected (Fig. 4a), although pKIT [Y721], [Y730], and [Y936] were undetectable by our immunofluorescence staining (data not shown). Interestingly, compared with KIT localization to EL, pKIT [Y703] was restricted to the perinuclear region in Kasumi-1 (Fig. 4a, top panels, arrowheads). Similar to KIT in Kasumi-1, KIT in HMC-1.1 was found in the perinuclear compartment (Fig. 4b). Perinuclear KIT autophosphorylation was colocalized with GM130 (Golgi) rather than with PDI (ER), TFR (endosomes), or LAMP1 (lysosomes) (Fig. 4c; Additional file 1: Figure S2A). Similar results were obtained with SKNO-1 (Additional file 1: Figure S2B & C). These results suggest that in leukemia cells, activation of KIT^N822K^ and KIT^V560G^ occurs predominantly on the Golgi although KIT itself is found mainly in EL.

### KIT^N822K^ and KIT^V560G^ mainly activate downstream molecules on the Golgi in leukemia cells

**Fig. 5:**
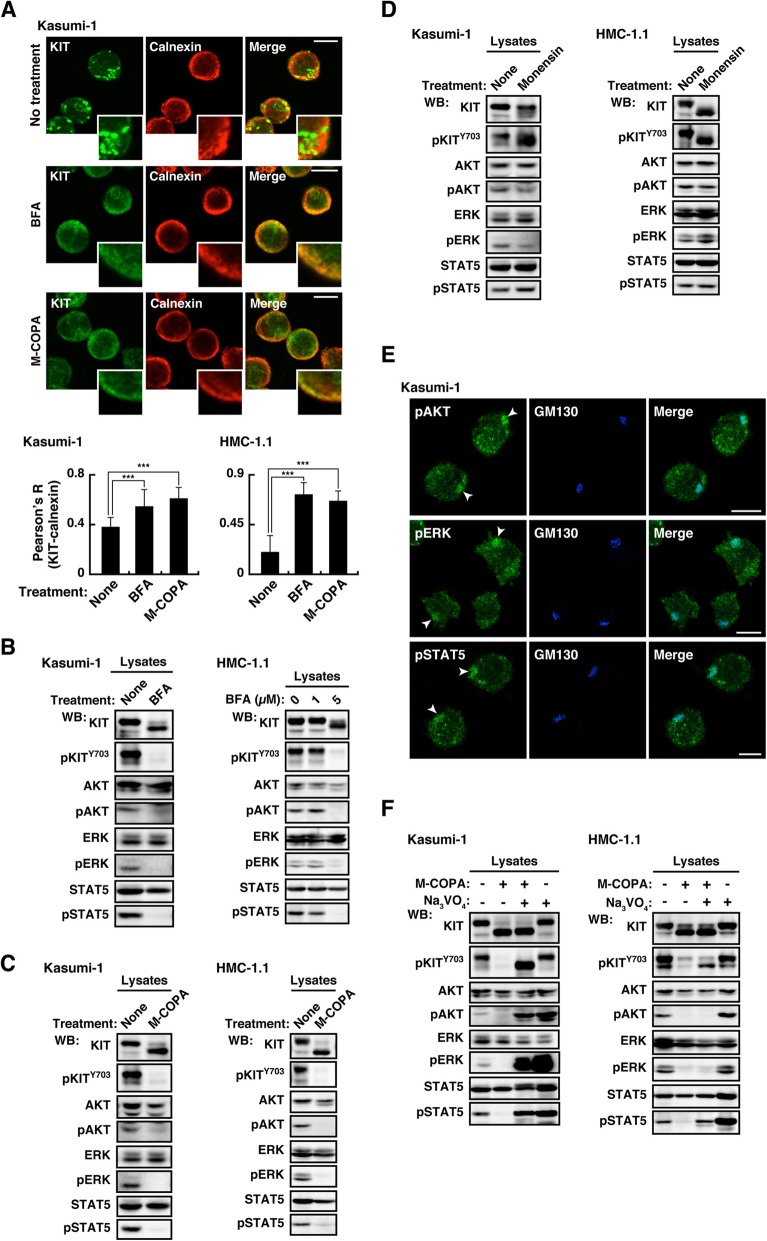
In Kasumi-1 and HMC-1.1, KIT activates downstream pathways on the Golgi apparatus. **a** Kasumi-1 cells were cultured for 12 h in the presence of 1 μM BFA or 1 μM M-COPA (inhibitors of ER export to the Golgi) and immunostained for KIT and calnexin (ER marker, red). Bars, 10 μm. Pearson’s R correlation coefficients were calculated by analyzing the intensity of KIT vs. calnexin. The right graph shows Pearson’s R (KIT-calnexin) for HMC-1.1 cells treated with 5 μM BFA or 1 μM M-COPA for 16 h. Results are means ± s.d. (*n* = 14~20). ***P < 0.001. **b**-**d** Kasumi-1 cells were treated for 12 h with 1 μM BFA, 1 μM M-COPA, or 250 nM monensin (an inhibitor of Golgi export to the PM). HMC-1.1 cells were treated with 1~5 μM BFA, 1 μM M-COPA for 16 h, or 250 nM monensin for 24 h. Lysates were immunoblotted. **e** Kasumi-1 cells were immunostained for phospho-AKT (pAKT, green), pERK (green), pSTAT5 (green), and GM130 (Golgi marker, blue). Bars, 10 μm. Arrowheads indicate the Golgi region. **f** Cells were treated with 1 μM M-COPA for 12 h (Kasumi-1) or 16 h (HMC-1.1), including 3 mM Na_3_VO_4_ (a PTP inhibitor) during the last 3 h, then immunoblotted

We then examined whether KIT activated downstream molecules on the Golgi in leukemia cells. To resolve this question, we used inhibitors of intracellular trafficking, such as brefeldin A (BFA), 2-methylcoprophilinamide (M-COPA) (inhibitors of ER export to the Golgi) [27, 34, 35, 41], monensin (an inhibitor of *intra*-Golgi trafficking) [26, 42], and bafilomycin A1 (BafA1, an inhibitor of endosome-to-lysosome trafficking) [24, 43]. In Kasumi-1 and HMC-1.1, treatment with BFA or M-COPA significantly increased colocalization of KIT with an ER marker, calnexin (Fig. 5a & Additional file 1: Figure S3A), confirming that the treatment inhibited ER export of KIT. Immunoblotting showed that KIT shifted to a lower molecular weight in BFA- or M-COPA-treated cells because of a defect in full glycosylation on the Golgi apparatus (Fig. 5b & c, top panels). KIT on the ER was dephosphorylated and unable to activate downstream molecules (Fig. 5b & c). Previous studies showed that a major target of BFA/M-COPA is Golgi-specific BFA-resistance guanine nucleotide exchange factor 1 (GBF1) that plays a role in the secretory pathway through activation of ADP ribosylation factor 1 (ARF1) [34, 44, 45]. Interestingly, knockdown (KD) of *ARF1* and *GBF1* with siRNAs did not cause a defect in full glycosylation of KIT or inhibition of signaling (Additional file 1: Figure S3B). Since BFA and M-COPA bind not only to the ARF1-GBF1 complex but also to other complexes [44, 45], the blockers affect KIT trafficking in a manner independent of ARF1-GBF1 inhibition in the leukemia cells used in this study. Further study will be required for understanding how the inhibitors block KIT trafficking from the ER.

Fig. 5d shows that inhibition of the Golgi export of KIT through blocking *intra*-Golgi trafficking did not suppress KIT signaling, suggesting that Golgi-localized KIT is sufficient for oncogenic signaling in Kasumi-1 and HMC-1.1. As shown in Additional file 1: Figure S3C, KIT signals remained in BafA1-treated cells, indicating that endosome-to-lysosome trafficking is unnecessary for downstream activation. Taken together, these results suggest that the Golgi apparatus serves as the platform for KIT activation in leukemia cells. To support this conclusion, we stained for phospho-AKT (pAKT), pERK, and pSTAT5. As shown in Fig. 5e, these phosphorylations were found at the Golgi region in Kasumi-1 cells. Compared with pAKT, total AKT was barely seen in the Golgi (Additional file 1: Figure S3D, upper panels). In Kasumi-1, only part of AKT may be activated by KIT. Furthermore, total ERK and STAT5 were distributed in the Golgi region (Additional file 1: Figure S3D). These results support our hypothesis that KIT activates these downstream molecules on the Golgi in leukemia cells. In HMC-1.1, AKT, ERK, STAT5, and their phospho-forms showed a diffuse distribution compared with those in Kasumi-1 (Additional file 1: Figure S3E). Since pAKT, pERK, pSTAT5, though small, were found in the Golgi region, they could be activated on the Golgi and subsequently move elsewhere.

Recently, we showed that in GISTs, KIT on the ER is dephosphorylated by protein tyrosine phosphatases (PTPs) [27]. We then considered the role of PTPs in KIT inactivation in the ER in leukemia cells. In M-COPA-pretreated Kasumi-1, a 3-h treatment with a PTP inhibitor (sodium orthovanadate, Na_3_VO_4_) [46] restored pKIT [Y703], resulting in downstream reactivation (Fig. 5f, left), indicating that in Kasumi-1, PTPs play a role in KIT inactivation in the ER. In M-COPA-treated HMC-1.1, pKIT [Y703] and pSTAT5 were recovered by Na_3_VO_4_ treatment, but AKT and ERK did not become active on PTP inhibition (Fig. 5f, right). Negative regulation of AKT and ERK may differ among cell types. Taken together, these results suggest that ER-localized KIT is inactivated by PTPs. PTP1B, Src homology 2 containing PTP-1 (SHP-1), and SHP-2 have been reported as PTPs for KIT and FLT3 RTKs [47–50]. Thus, we knocked down these *PTPs* and treated cells with M-COPA to investigate the key PTPs for KIT in the ER. Additional file 1: Figure S4 shows that in M-COPA-treated cells, pKIT [Y703], pAKT, and pERK were not restored by *PTP1B* or *SHP1/2* KD, suggesting that these PTPs are not responsible for this dephosphorylation in the ER. Interestingly, *PTP1B* but not *SHP1/2* KD partially rescued pSTAT5 in M-COPA-treated cells (Additional file 1: Figure S4A, arrows). Although we were unable to identify KIT phospho-tyrosine sites that are dephosphorylated by PTP1B in this study, PTP1B in the ER may play a role in inactivation of the KIT-STAT5 axis.

SKNO-1 cells were similar to Kasumi-1 in phospho-regulation of KIT in intracellular compartments (Additional file 1: Figure S5A & B). However, AKT, ERK, and STAT5 were not activated by KIT^N822K^ (Additional file 1: Figure S5C). SKNO-1 requires GM-CSF for proliferation, but the cytokine did not affect the activation of KIT, AKT, ERK, or STAT5 (Additional file 1: Figure S5C, right panels). AKT and ERK were not found in specific compartments in SKNO-1 in the presence or absence of GM-CSF (Additional file 1: Figure S5D). At present, we are unable to find downstream molecules that are activated by KIT^N822K^ in SKNO-1 cells. We will investigate the role of KIT^N822K^ in SKNO-1 growth in the near future.

### Lipid rafts play a key role in KIT signaling, which occurs on the Golgi apparatus

**Fig. 6:**
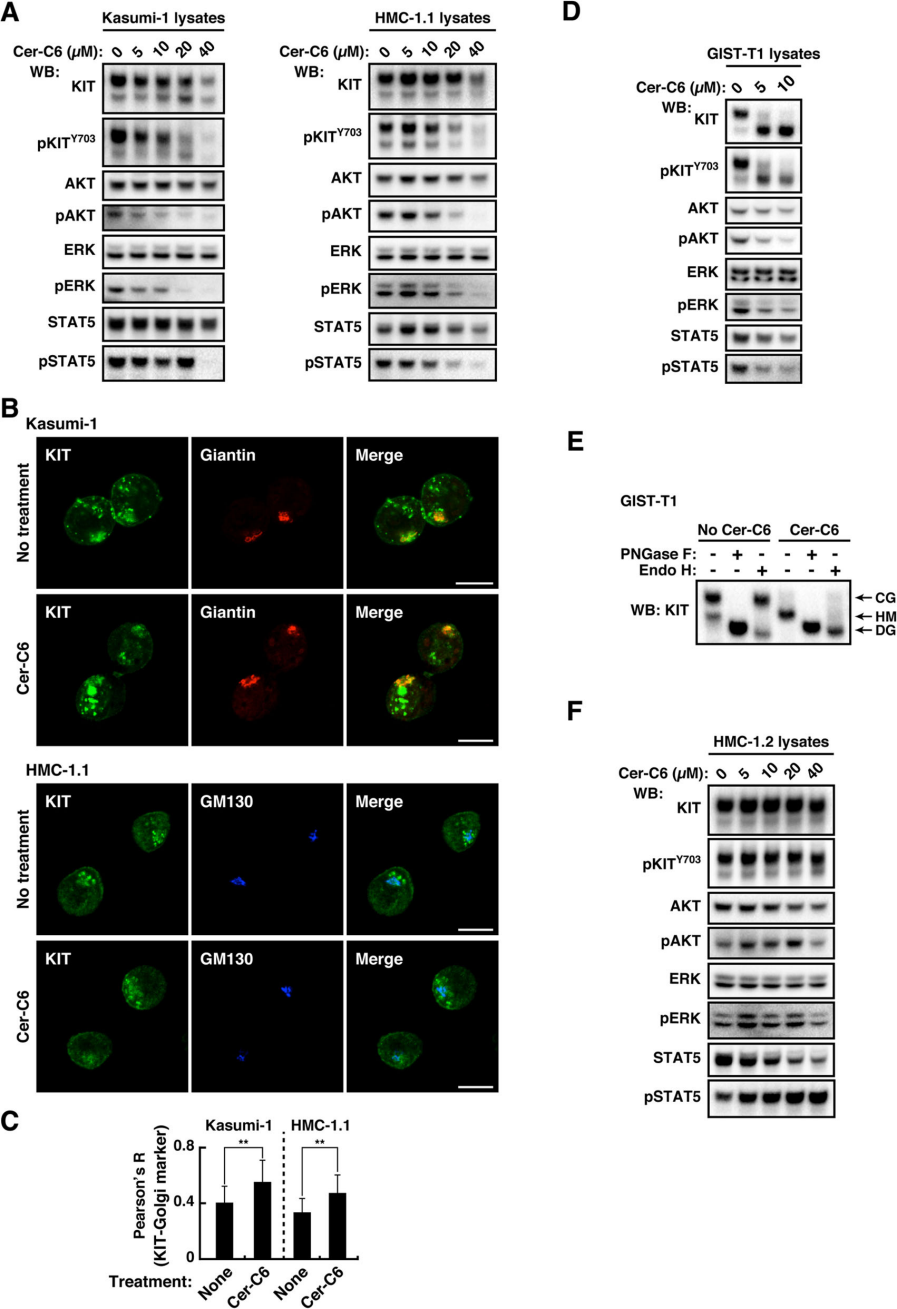
Lipid rafts have a role in KIT signaling at the Golgi apparatus. **a**-**c** Kasumi-1 or HMC-1.1 cells were treated with 0~40 μM cer-C6 for 8 h (for inhibition of normal lipid raft formation). **a** Lysates were immunoblotted. **b** Cells treated with 40 μM cer-C6 for 8 h were immunostained for KIT (green), giantin (Golgi marker, red), or GM130 (Golgi marker, blue). Bars, 10 μm. **c** Pearson’s R correlation coefficients were calculated by analyzing the intensity of KIT vs. giantin (Kasumi-1) or GM130 (HMC-1.1). Results are means ± s.d. (*n* = 16~22). **P < 0.01. **d** & **e** GIST-T1 cells were treated with 0~10 μM cer-C6 for 10 h. **d** Lysates were immunoblotted. **e** Lysates were treated with PNGase F or endoglycosidase H then immunoblotted with anti-KIT. CG, complex-glycosylated form; HM, high mannose form; DG, deglycosylated form. **f** HMC-1.2 cells were treated with 0~40 μM cer-C6 for 8 h, then immunoblotted

Recent studies showed that sphingomyelin-enriched membrane microdomains (lipid rafts) in the Golgi are needed for activation of an innate immunity molecule, STING [51, 52]. Formation of normal lipid microdomains is inhibited by N-hexanoyl-D-erythro-sphingosine (cer-C6) through producing short chain sphingomyelin that disrupts the lipid order [51, 53, 54]. Figure 6a shows that in Kasumi-1, cer-C6 treatment lowered the protein levels of KIT and inhibited KIT autophosphorylation and the activation of AKT, ERK, and STAT5 in a dose-dependent manner. The treatment did not decrease but rather increased KIT on the Golgi (Fig. 6b & c). These results suggest that KIT^N822K^ and KIT^V560G^ require lipid rafts for their stability and activation in the Golgi in these leukemia cells.

Finally, we asked whether lipid rafts play a role in oncogenic signaling by all KIT mutants. GIST-T1 cells (*KIT*^*⊿560–578*^) grow in a manner dependent on KIT signaling on the Golgi, whereas HMC-1.2 (mast cell leukemia, *KIT*^*V560G/D816V*^) requires pAKT on EL and pSTAT5 on the ER [24–27] (Additional file 1: Figure S6A & Table 1). In both cell types, TKIs increased PM distribution of KIT mutants (Additional file 1: Figure S6B), supporting our data obtained with Kasumi-1 that mutant KIT localizes to intracellular compartments in a manner dependent on its kinase activity. In GIST-T1, cer-C6 inhibited the phosphorylation of KIT and downstream molecules (Fig. 6d). Unlike KIT in leukemia cells, that in cer-C6-treated GIST-T1 assumed an immature glycosylated form (Fig. 6e), indicating that KIT is complex-glycosylated in GIST after reaching lipid rafts. Similar to the results using Kasumi-1, the treatment did not decrease but rather increased KIT on the Golgi (Additional file 1: Figure S6C). On the other hand, in HMC-1.2, cer-C6 did not have an inhibitory effect on Golgi export of KIT^D816V^ and growth signals (Fig. 6f; Additional file 1: Figure S6D). Therefore, lipid rafts play a critical role in KIT signaling that occurs on the Golgi.

## Discussion

**Fig. 7:**
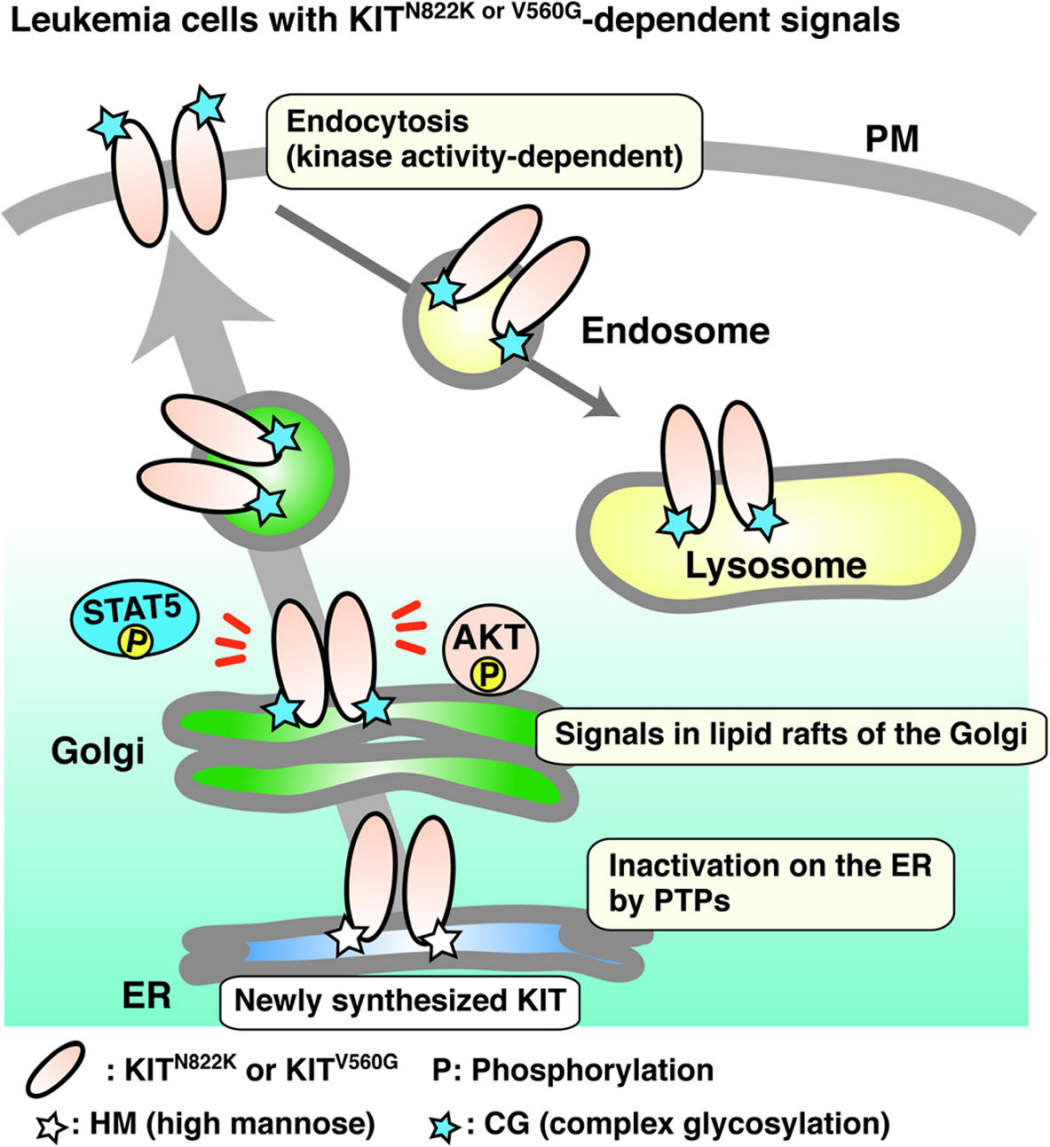
Model of mutant KIT trafficking and signals on the Golgi in leukemia cells. Newly synthesized mutant KIT (KIT^N822K^ or KIT^V560G^) in the ER traffics to the PM through the Golgi apparatus. They are normally complex-glycosylated in the Golgi. After reaching the PM, mutant KIT immediately undergoes endocytosis in a manner dependent on its kinase activity, then accumulates in EL. However, its full autophosphorylation mainly occurs on the Golgi, where it causes downstream activation. Lipid rafts play a role in KIT signaling. ER-localized KIT is inactivated by PTPs

In this study, we demonstrate that in leukemia cells, localization of KIT^N822K^ and KIT^V560G^ is clearly different from that of KIT^WT^ in normal cells. We provide evidence that after secretory trafficking to the PM, these mutants localize to EL through kinase activity-dependent endocytosis. However, they are autophosphorylated predominantly on the Golgi apparatus, where they activate downstream molecules, such as AKT, ERK, and STAT5. Lipid rafts play a key role in KIT signaling on the Golgi. Moreover, ER-localized KIT is dephosphorylated by PTPs (Fig. 7). This phospho-regulation of KIT is similar to that in GISTs. Our observations show that in some cases, receptor mutants can initiate signals from the Golgi even if they are mainly present in EL (Table 1).

Recently, we reported that in MCL, AL-mut, such as KIT^D816V^ (human) and KIT^D814Y^ (mouse), accumulates in EL (Table 1) [24, 25]. Unlike JM-mut, AL-mut in MCL activates AKT and STAT5 on EL and the ER, respectively. Previous studies showed that transfected AL-mut in cell lines other than MCL, such as NIH3T3 and GISTs, localizes to the Golgi, where it initiates oncogenic signals on the Golgi apparatus (Table 1) [26, 28]. The host cell environment rather than the KIT mutation status may determine the mutant’s subcellular localization. Considering that JM-mut in MCL activates a downstream pathway on the Golgi, these studies suggest that AL-mut activates downstream molecules on EL and the ER only when it is expressed in an MCL environment. Moreover, there is great interest in further investigation as to whether KIT^D816V^ in AML causes growth signaling on the ER, Golgi, or EL. Further studies will be required for understanding the mechanism responsible for the difference in the signal platforms of KIT.

In addition to previous reports on MCL and GISTs [24, 26, 30], this study suggests that KIT^N822K^ also mis-localizes to intracellular organelles in AML. Permanently active KIT mutations are found in about 25% of mucosal melanomas and seminomas [22, 55–57]. Thus, we will examine the relationship between KIT localization and oncogenic signaling in these cancers in the near future. Furthermore, recent studies reported that cancer-causing receptor mutants and splice variants also accumulate on intracellular compartments in an aberrant manner [58]. FLT3-internal tandem duplication (FLT3-ITD, AML), FGFR3^K650E^ (multiple myeloma), PDGFRA^Y288C or V561D^ (carcinomas/GIST), and a splice variant of TRKA (TRKAIII, neuroblastoma) are found in early secretory compartments [59–65], and EGFR^L858R/T790M^, GP130^⊿YY^, and CSF3R^T618I^ mislocalize to endosomes [66–68]. These reports and the findings of our study raised the intriguing possibility that mislocalization in cancer cells is a ubiquitous feature of aberrant receptors.

In innate immune cells, STING binds to exogenous DNA fragments in the ER, then moves to lipid rafts of the Golgi [51, 52]. This requires palmitoylation of its cysteine residues for migration to the lipid rafts, where it can activate the TBK1-IRF3 pathway [51]. MET tyrosine kinase is activated and palmitoylated on its extracellular domain at the Golgi [69, 70], and lipid rafts contribute to receptor distribution and stability [70, 71]. These studies raise the interesting possibility that incorporation of KIT into the lipid rafts of the Golgi is involved in protein acylation. Investigation as to whether palmitoylation is necessary for KIT signaling on the Golgi is now under way.

PTPs play a role in inactivation of KIT in the ER. Our loss of function study showed that PTP1B, SHP-1, and SHP-2 are not major PTPs for KIT dephosphorylation in the ER, suggesting the role of other PTPs in KIT inactivation. In addition, negative regulators of KIT and downstream molecules could be abundant in intracellular compartments other than the Golgi. Further analyses of the localization and functions of these negative regulators should explain how KIT signaling is inactivated in the ER, PM, and EL both in leukemia and GISTs. In other words, the study will reveal the mechanism of deregulation of RTK on signal platforms.

In this study, KIT mutants were retained in the PM in TKI-treated cells, since TKIs inhibit endocytosis of KIT, which depends on the kinase activity. Furthermore, TKIs increase PM localization of EGFR in lung cancers and PDGFRA/KIT in GISTs [26, 63, 72]. A previous report showed that TKI treatment increases the FLT3-ITD PM level in AML, which enhances the effect of FLT3-directed immunotherapy in mice [73]. Moreover, anti-KIT antibody is efficacious for suppression of the autonomous growth of GIST cells [74, 75]. From a clinical point of view, combined therapy with anti-RTK antibodies and TKIs seems attractive.

Small molecule TKIs and antibodies against RTKs have been developed for suppression of proliferative signals in cancers. In this study, blockade of the ER export of KIT with BFA/M-COPA decreased KIT’s autophosphorylation in leukemia cells. Together with the results previous reports [25, 27, 76, 77], our findings may offer a trafficking blockade of receptor mutants as a third strategy for inhibition of oncogenic signaling.

## Conclusions

In conclusion, we show that in leukemia cells, N822K- and V560G-mutated KIT can initiate growth signals in lipid rafts of the Golgi apparatus. These observations provide new insights into the pathogenic role of KIT mutants as well as into that of other mutant signaling molecules. Moreover, from a clinical point of view, our findings offer a new strategy for leukemia treatment through that blocks the incorporation of KIT mutants into the lipid rafts of the Golgi.

## Additional file

Additional file 1: **Figure S1.** Mutant KIT localizes preferentially to EL in HMC-1.1 and SKNO-1 cells. **Figure S2.** In HMC-1.1 and SKNO-1, the major site for KIT autophosphorylation is colocalized with the Golgi region. **Figure S3.** Distribution of signal molecules in Kasumi-1 and HMC-1.1 cells. **Figure S4.** Effect of knockdown of *PTP1B*, *SHP-1*, and *SHP-2* on KIT signals. **Figure S5.** KIT^N822K^ does not activate AKT, ERK, and STAT5 in SKNO-1 cells. **Figure S6.** Effect of inhibition of normal lipid raft formation on KIT distribution. (PDF 14400 kb)

## Abbreviations

AML: Acute myeloid leukemia
BafA1: Bafilomycin A1
BFA: Brefeldin A
Cer-C6: N-hexanoyl-D-erythro-sphingosine
EL: Endosome-lysosomal compartments
ER: Endoplasmic reticulum
ERK: Extracellular signal-regulated kinase;
FLT3: Fms-like tyrosine kinase 3
GIST: Gastrointestinal stromal tumor
MCL: Mast cell leukemia
M-COPA: 2-methylcoprophilinamide
mut: Mutant
PDGFR: Platelet-derived growth factor receptor
pKIT: Phospho-KIT
PM: Plasma membrane
PTP: Protein tyrosine phosphatase
RTK: Receptor tyrosine kinase
SCF: Stem cell factor
STAT: Signal transducer and activator of transcription
TKI: Tyrosine kinase inhibitor
wt: Wild-type

## References

[1] Besmer Peter, Murphy John E., George Patricia C., Qiu Feihua, Bergold Peter J., Lederman Lynn, Snyder Harry W., Brodeur David, Zuckerman Evelyn E., Hardy William D. (1986). A new acute transforming feline retrovirus and relationship of its oncogene v-kit with the protein kinase gene family. Nature.

[2] Yarden Y., Kuang W. J., Yang-Feng T., Coussens L., Munemitsu S., Dull T. J., Chen E., Schlessinger J., Francke U., Ullrich A. (1987). Human proto-oncogene c-kit: a new cell surface receptor tyrosine kinase for an unidentified ligand. The EMBO Journal.

[3] Blume-Jensen Peter, Hunter Tony (2001). Oncogenic kinase signalling. Nature.

[4] Broudy VC. Stem cell factor and hematopoiesis. Blood. 1997;90:1345–64. 9269751

[5] Thomson Lars, Robinson Tim L., Lee Jonathan C.F., Farraway Laura A., Hughes Martin J.G., Andrews David W., Huizinga Jan D. (1998). Interstitial cells of Cajal generate a rhythmic pacemaker current. Nature Medicine.

[6] Lennartsson Johan, Rönnstrand Lars (2012). Stem Cell Factor Receptor/c-Kit: From Basic Science to Clinical Implications. Physiological Reviews.

[7] Roskoski Robert (2005). Structure and regulation of Kit protein-tyrosine kinase—The stem cell factor receptor. Biochemical and Biophysical Research Communications.

[8] THÖMMES Kerstin, LENNARTSSON Johan, CARLBERG Monika, RÖNNSTRAND Lars (1999). Identification of Tyr-703 and Tyr-936 as the primary association sites for Grb2 and Grb7 in the c-Kit/stem cell factor receptor. Biochemical Journal.

[9] Blume-Jensen Peter, Janknecht Ralf, Hunter Tony (1998). The Kit receptor promotes cell survival via activation of PI 3-kinase and subsequent Akt-mediated phosphorylation of Bad on Ser136. Current Biology.

[10] Blume-Jensen P., Claesson-Welsh L., Siegbahn A., Zsebo K.M., Westermark B., Heldin C.H. (1991). Activation of the human c-kit product by ligand-induced dimerization mediates circular actin reorganization and chemotaxis. The EMBO Journal.

[11] Hong L., Munugalavadla V., Kapur R. (2004). c-Kit-Mediated Overlapping and Unique Functional and Biochemical Outcomes via Diverse Signaling Pathways. Molecular and Cellular Biology.

[12] Chan P. M., Ilangumaran S., La Rose J., Chakrabartty A., Rottapel R. (2003). Autoinhibition of the Kit Receptor Tyrosine Kinase by the Cytosolic Juxtamembrane Region. Molecular and Cellular Biology.

[13] Hirota S. (1998). Gain-of-Function Mutations of c-kit in Human Gastrointestinal Stromal Tumors. Science.

[14] Boissan M, Feger F, Guillosson JJ, Arock M (2000). C-kit and c-kit mutations in mastocytosis and other hematological diseases. J Leukoc Biol.

[15] Baird JH, Gotlib J (2018). Clinical validation of KIT inhibition in advanced systemic mastocytosis. Curr Hematol Malig Rep.

[16] Kitamura Y, Hirota S (2004). Kit as a human oncogenic tyrosine kinase. Cell Mol Life Sci.

[17] Corless CL, Barnett CM, Heinrich MC (2011). Gastrointestinal stromal tumours: origin and molecular oncology. Nat Rev Cancer.

[18] Lasota J, Miettinen M (2008). Clinical significance of oncogenic *KIT* and *PDGFRA* mutations in gastrointestinal stromal tumours. Histopathology..

[19] De Giorgi U, Verweij J (2005). Imatinib and gastrointestinal stromal tumors: where do we go from here?. Mol Cancer Ther.

[20] Joensuu H, Roberts PJ, Sarlomo-Rikala M, Andersson LC, Tervahartiala P, Tuveson D, Silberman S, Capdeville R, Dimitrijevic S, Druker B (2001). Effect of the tyrosine kinase inhibitor STI571 in a patient with a metastatic gastrointestinal stromal tumor. N Engl J Med.

[21] Frost MJ, Ferrao PT, Hughes TP, Ashman LK (2002). Juxtamembrane mutant V560GKit is more sensitive to Imatinib (STI571) compared with wild-type c-kit whereas the kinase domain mutant D816VKit is resistant. Mol Cancer Ther.

[22] Kemmer K, Corless CL, Fletcher JA, McGreevey L, Haley A, Griffith D, Cummings OW, Wait C, Town A, Heinrich MC (2004). KIT mutations are common in testicular seminomas. Am J Pathol.

[23] Omori I, Yamaguchi H, Miyake K, Miyake N, Kitano T, Inokuchi K (2017). D816V mutation in the KIT gene activation loop has greater cell-proliferative and anti-apoptotic ability than N822K mutation in core-binding factor acute myeloid leukemia. Exp Hematol.

[24] Obata Y, Toyoshima S, Wakamatsu E, Suzuki S, Ogawa S, Esumi H, Abe R (2014). Oncogenic kit signals on endolysosomes and endoplasmic reticulum are essential for neoplastic mast cell proliferation. Nat Commun.

[25] Hara Yasushi, Obata Yuuki, Horikawa Keita, Tasaki Yasutaka, Suzuki Kyohei, Murata Takatsugu, Shiina Isamu, Abe Ryo (2017). M-COPA suppresses endolysosomal Kit-Akt oncogenic signalling through inhibiting the secretory pathway in neoplastic mast cells. PLOS ONE.

[26] Obata Y, Horikawa K, Takahashi T, Akieda Y, Tsujimoto M, Fletcher JA, Esumi H, Nishida T, Abe R (2017). Oncogenic signaling by kit tyrosine kinase occurs selectively on the Golgi apparatus in gastrointestinal stromal tumors. Oncogene..

[27] Obata Y, Horikawa K, Shiina I, Takahashi T, Murata T, Tasaki Y, Suzuki K, Yonekura K, Esumi H, Nishida T (2018). Oncogenic kit signalling on the Golgi is suppressed by blocking secretory trafficking with M-COPA in GISTs. Cancer Lett.

[28] Xiang Z, Kreisel F, Cain J, Colson AL, Tomasson MH (2007). Neoplasia driven by mutant *c-KIT* is mediated by intracellular, not plasma membrane, receptor signaling. Mol Cell Biol.

[29] Kim WK, Yun S, Park CK, Bauer S, Kim J, Lee MG, Kim H (2017). Sustained mutant KIT activation in the Golgi complex is mediated by PKC-θ in gastrointestinal stromal tumors. Clin Cancer Res.

[30] Tabone-Eglinger S, Subra F, El Sayadi H, Alberti L, Tabone E, Michot JP, Théou-Anton N, Lemoine A, Blay JY, Emile JF (2008). *KIT* mutations induce intracellular retention and activation of an immature form of the KIT protein in gastrointestinal stromal tumors. Clin Cancer Res.

[31] Wakita S, Yamaguchi H, Miyake K, Mitamura Y, Kosaka F, Dan K, Inokuchi K (2011). Importance of c-kit mutation detection method sensitivity in prognostic analyses of t (8;21)(q22;q22) acute myeloid leukemia. Leukemia..

[32] Yui S, Kurosawa S, Yamaguchi H, Kanamori H, Ueki T, Uoshima N, Mizuno I, Shono K, Usuki K, Chiba S (2017). D816 mutation of the KIT gene in core binding factor acute myeloid leukemia is associated with poorer prognosis than other KIT gene mutations. Ann Hematol.

[33] Tarlock Katherine, Alonzo Todd A., Wang Yi-Cheng, Gerbing Robert B., Ries Rhonda, Loken Michael R., Pardo Laura, Hylkema Tiffany, Joaquin Jason, Sarukkai Leela, Raimondi Susana C., Hirsch Betsy, Sung Lillian, Aplenc Richard, Bernstein Irwin, Gamis Alan S., Meshinchi Soheil, Pollard Jessica A. (2019). Functional Properties of KIT Mutations Are Associated with Differential Clinical Outcomes and Response to Targeted Therapeutics in CBF Acute Myeloid Leukemia. Clinical Cancer Research.

[34] Shiina I, Umezaki Y, Ohashi Y, Yamazaki Y, Dan S, Yamori T (2013). Total synthesis of AMF-26, an antitumor agent for inhibition of the Golgi system, targeting ADP-ribosylation factor 1. J Med Chem.

[35] Shiina I, Umezaki Y, Murata T, Suzuki K, Tonoi T (2018). Asymmetric total synthesis of (+)-coprophilin. Synthesis..

[36] Aichberger KJ, Gleixner KV, Mirkina I, Cerny-Reiterer S, Peter B, Ferenc V, Kneidinger M, Baumgartner C, Mayerhofer M, Gruze A (2009). Identification of proapoptotic Bim as a tumor suppressor in neoplastic mast cells: role of KIT D816V and effects of various targeted drugs. Blood..

[37] Fang HT, Zhang B, Pan XF, Gao L, Zhen T, Zhao HX, Ma L, Xie J, Liu Z, Yu XJ (2012). Bortezomib interferes with C-KIT processing and transforms the t (8;21)-generated fusion proteins into tumor-suppressing fragments in leukemia cells. Proc Natl Acad Sci U S A.

[38] Gleixner KV, Peter B, Blatt K, Suppan V, Reiter A, Radia D, Hadzijusufovic E, Valent P (2013). Synergistic growth-inhibitory effects of ponatinib and midostaurin (PKC412) on neoplastic mast cells carrying KIT D816V. Haematologica..

[39] Chen LT, Chen CT, Jiaang WT, Chen TY, Butterfield JH, Shih NY, Hsu JT, Lin HY, Lin SF, Tsai HJ (2016). BPR1J373, an oral multiple tyrosine kinase inhibitor, targets c-KIT for the treatment of c-KIT-driven myeloid leukemia. Mol Cancer Ther.

[40] Bougherara H, Subra F, Crépin R, Tauc P, Auclair C, Poul MA (2009). The aberrant localization of oncogenic kit tyrosine kinase receptor mutants is reversed on specific inhibitory treatment. Mol Cancer Res.

[41] Klausner R. D. (1992). Brefeldin A: insights into the control of membrane traffic and organelle structure. The Journal of Cell Biology.

[42] Griffiths G, Quinn P, Warren G (1983). Dissection of the Golgi complex. I. Monensin inhibits the transport of viral membrane proteins from *medial* to *trans* Golgi cisternae in baby hamster kidney cells infected with Semliki Forest virus. J Cell Biol.

[43] Brown D, Paunescu TG, Breton S, Marshansky V (2009). Regulation of the V-ATPase in kidney epithelial cells: dual role in acid-base homeostasis and vesicle trafficking. J Exp Biol.

[44] Niu TK, Pfeifer AC, Lippincott-Schwartz J, Jackson CL (2005). Dynamics of GBF1, a Brefeldin A-sensitive Arf1 exchange factor at the Golgi. Mol Biol Cell.

[45] Ignashkova TI, Gendarme M, Peschk K, Eggenweiler HM, Lindemann RK, Reiling JH (2017). Cell survival and protein secretion associated with Golgi integrity in response to Golgi stress-inducing agents. Traffic..

[46] Yonemoto W, Filson AJ, Queral-Lustig AE, Wang JY, Brugge JS (1987). Detection of phosphotyrosine-containing proteins in polyomavirus middle tumor antigen-transformed cells after treatment with a phosphotyrosine phosphatase inhibitor. Mol Cell Biol.

[47] Julien SG, Dubé N, Hardy S, Tremblay MN (2011). Inside the human cancer tyrosine phosphatome. Nat Rev Cancer.

[48] Kozlowski M, Larose L, Lee F, Le DM, Rottapel R, Siminovitch KA (1998). SHP-1 binds and negatively modulates the c-kit receptor by interaction with tyrosine 569 in the c-kit juxtamembrane domain. Mol Cell Biol.

[49] Yu M, Luo J, Yang W, Wang Y, Mizuki M, Kanakura Y, Besmer P, Neel BG, Gu H (2006). The scaffolding adapter Gab2, via Shp-2, regulates kit-evoked mast cell proliferation by activating the Rac/JNK pathway. J Biol Chem.

[50] Schmidt-Arras DE, Böhmer A, Markova B, Choudhary C, Serve H, Böhmer FD (2005). Tyrosine phosphorylation regulates maturation of receptor tyrosine kinases. Mol Cell Biol.

[51] Mukai K, Konno H, Akiba T, Uemura T, Waguri S, Kobayashi T, Barber GN, Arai H, Taguchi T (2016). Activation of STING requires palmitoylation at the Golgi. Nat Commun.

[52] Haag SM, Gulen MF, Reymond L, Gibelin A, Abrami L, Decout A, Heymann M, van der Goot FG, Turcatti G, Behrendt R (2018). Targeting STING with covalent small-molecule inhibitors. Nature..

[53] van Galen J, Campelo F, Martínez-Alonso E, Scarpa M, Martínez-Menárguez JÁ, Malhotra V (2014). Sphingomyelin homeostasis is required to form functional enzymatic domains at the *trans*-Golgi network. J Cell Biol.

[54] Duran JM, Campelo F, van Galen J, Sachsenheimer T, Sot J, Egorov MV, Rentero C, Enrich C, Polishchuk RS, Goñi FM (2012). Sphingomyelin organization is required for vesicle biogenesis at the Golgi complex. EMBO J.

[55] Hayward NK, Wilmott JS, Waddell N, Johansson PA, Field MA, Nones K, Patch AM, Kakavand H, Alexandrov LB, Burke H (2017). Whole-genome landscapes of major melanoma subtypes. Nature..

[56] Shen H, Shih J, Hollern DP, Wang L, Bowlby R, Tickoo SK, Thorsson V, Mungall AJ, Newton Y, Hegde AM (2018). Integrated molecular characterization of testicular germ cell tumors. Cell Rep.

[57] Newell F, Kong Y, Wilmott JS, Johansson PA, Ferguson PM, Cui C, Li Z, Kazakoff SH, Burke H, Dodds TJ (2019). Whole-genome landscape of mucosal melanoma reveals diverse drivers and therapeutic targets. Nat Commun.

[58] Toffalini F, Demoulin JB (2010). New insights into the mechanisms of hematopoietic cell transformation by activated receptor tyrosine kinases. Blood..

[59] Köthe S, Müller JP, Böhmer SA, Tschongov T, Fricke M, Koch S, Thiede C, Requardt RP, Rubio I, Böhmer FD (2013). Features of Ras activation by a mislocalized oncogenic tyrosine kinase: FLT3 ITD signals through K-Ras at the plasma membrane of acute myeloid leukemia cells. J Cell Sci.

[60] Choudhary C, Olsen JV, Brandts C, Cox J, Reddy PN, Böhmer FD, Gerke V, Schmidt-Arras DE, Berdel WE, Müller-Tidow C (2009). Mislocalized activation of oncogenic RTKs switches downstream signaling outcomes. Mol Cell.

[61] Ronchetti D, Greco A, Compasso S, Colombo G, Dell'Era P, Otsuki T, Lombardi L, Neri A (2001). Deregulated FGFR3 mutants in multiple myeloma cell lines with t (4;14): comparative analysis of Y373C, K650E and the novel G384D mutations. Oncogene..

[62] Gibbs L, Legeai-Maller L (2007). FGFR3 intracellular mutations induce tyrosine phosphorylation in the Golgi and defective glycosylation. Biochim Biophys Acta.

[63] Bahlawane C, Eulenfeld R, Wiesinger MY, Wang J, Muller A, Girod A, Nazarov PV, Felsch K, Vallar L, Sauter T, et al. Constitutive activation of oncogenic PDGFRα-mutant proteins occurring in GIST patients induces receptor mislocalisation and alters PDGFRα signalling characteristics. Cell Commun Signal. 2015;13. 10.1186/s12964-015-0096-8. 10.1186/s12964-015-0096-8 PMC4396151 25880691

[64] Ip CKM, Ng PKS, Jeong KJ, Shao SH, Ju Z, Leonard PG, Hua X, Vellano CP, Woessner R, Sahni N (2018). Neomorphic PDGFRA extracellular domain driver mutations are resistant to PDGFRA targeted therapies. Nat Commun.

[65] Farina AR, Cappabianca L, Ruggeri P, Gneo L, Maccarone R, Mackay AR (2015). Retrograde TrkAIII transport from ERGIC to ER: a re-localisation mechanism for oncogenic activity. Oncotarget..

[66] Chung BM, Raja SM, Clubb RJ, Tu C, George M, Band V (2009). Aberrant trafficking of NSCLC-associated EGFR mutants through the endocytic recycling pathway promotes interaction with Src. BMC Cell Biol.

[67] Schmidt-Arras D, Müller M, Stevanovic M, Horn S, Schütt A, Bergmann J, Wilkens R, Lickert A, Rose-John S (2014). Oncogenic deletion mutants of gp130 signal from intracellular compartments. J Cell Sci.

[68] Zhang H, Coblentz C, Watanabe-Smith K, Means S, Means J, Maxson JE, Tyner JW (2018). Gain-of-function mutations in granulocyte colony-stimulating factor receptor (CSF3R) reveal distinct mechanisms of CSF3R activation. J Biol Chem.

[69] Frazier Nicole Michael, Brand Toni, Gordan John D., Grandis Jennifer, Jura Natalia (2018). Overexpression-mediated activation of MET in the Golgi promotes HER3/ERBB3 phosphorylation. Oncogene.

[70] Coleman DT, Gray AL, Kridel SJ, Cardelli JA (2016). Palmitoylation regulates the intracellular trafficking and stability of c-met. Oncotarget..

[71] Zhu L, Xiong X, Kim Y, Okada N, Lu F, Zhang H, Sun H (2016). Acid sphingomyelinase is required for cell surface presentation of met receptor tyrosine kinase in cancer cells. J Cell Sci.

[72] Watanuki Z, Kosai H, Osanai N, Ogama N, Mochizuki M, Tamai K, Yamaguchi K, Satoh K, Fukuhara T, Maemondo M (2014). Synergistic cytotoxicity of afatinib and cetuximab against EGFR T790M involves Rab11-dependent EGFR recycling. Biochem Biophys Res Commun.

[73] Reiter K, Polzer H, Krupka C, Maiser A, Vick B, Rothenberg-Thurley M, Metzeler KH, Dörfel D, Salih HR, Jung G (2018). Tyrosine kinase inhibition increases the cell surface localization of FLT3-ITD and enhances FLT3-directed immunotherapy of acute myeloid leukemia. Leukemia..

[74] Edris B, Willingham SB, Weiskopf K, Volkmer AK, Volkmer JP, Mühlenberg T, Montgomery KD, Contreras-Trujillo H, Czechowicz A, Fletcher JA (2013). Anti-KIT monoclonal antibody inhibits imatinib-resistant gastrointestinal stromal tumor growth. Proc Natl Acad Sci U S A.

[75] Fujimoto S, Muguruma N, Okamoto K, Kurihara T, Sato Y, Miyamoto Y, Kitamura S, Miyamoto H, Taguchi T, Tsuneyama K (2018). A novel theranostic combination of near-infrared fluorescence imaging and laser irradiation targeting c-KIT for gastrointestinal stromal tumors. Theranostics..

[76] Williams AB, Li L, Nguyen B, Brown P, Levis M, Small D (2012). Fluvastatin inhibits FLT3 glycosylation in human and murine cells and prolongs survival of mice with FLT3/ITD leukemia. Blood..

[77] Zappa F, Failli M, De Matteis MA (2018). The Golgi complex in disease and therapy. Curr Opin Cell Biol.

