# Supplementary material for: N822K- or V560G-mutated KIT activation preferentially occurs in lipid rafts of the Golgi apparatus in leukemia cells
Source: Cell Commun Signal. 2019 Sep 4;17:114. doi: 10.1186/s12964-019-0426-3 (PMC6727407; doi:10.1186/s12964-019-0426-3)
Supplement: Supplementary file 1 — Figure S1. Mutant KIT localizes preferentially to EL in HMC-1.1 and SKNO-1 cells. Figure S2. In HMC-1.1 and SKNO-1, the major site for KIT autophosphorylation is colocalized with the Golgi region. Figure S3. Distribution of signal molecules in Kasumi-1 and HMC-1.1 cells. Figure S4. Effect of knockdown of PTP1B, SHP-1, and SHP-2 on KIT signals. Figure S5. KITN822K does not activate AKT, ERK, and STAT5 in SKNO-1 cells. Figure S6. Effect of inhibition of normal lipid raft formation on KIT distribution. (PDF 14400 kb) [file 12964_2019_426_MOESM1_ESM.pdf]

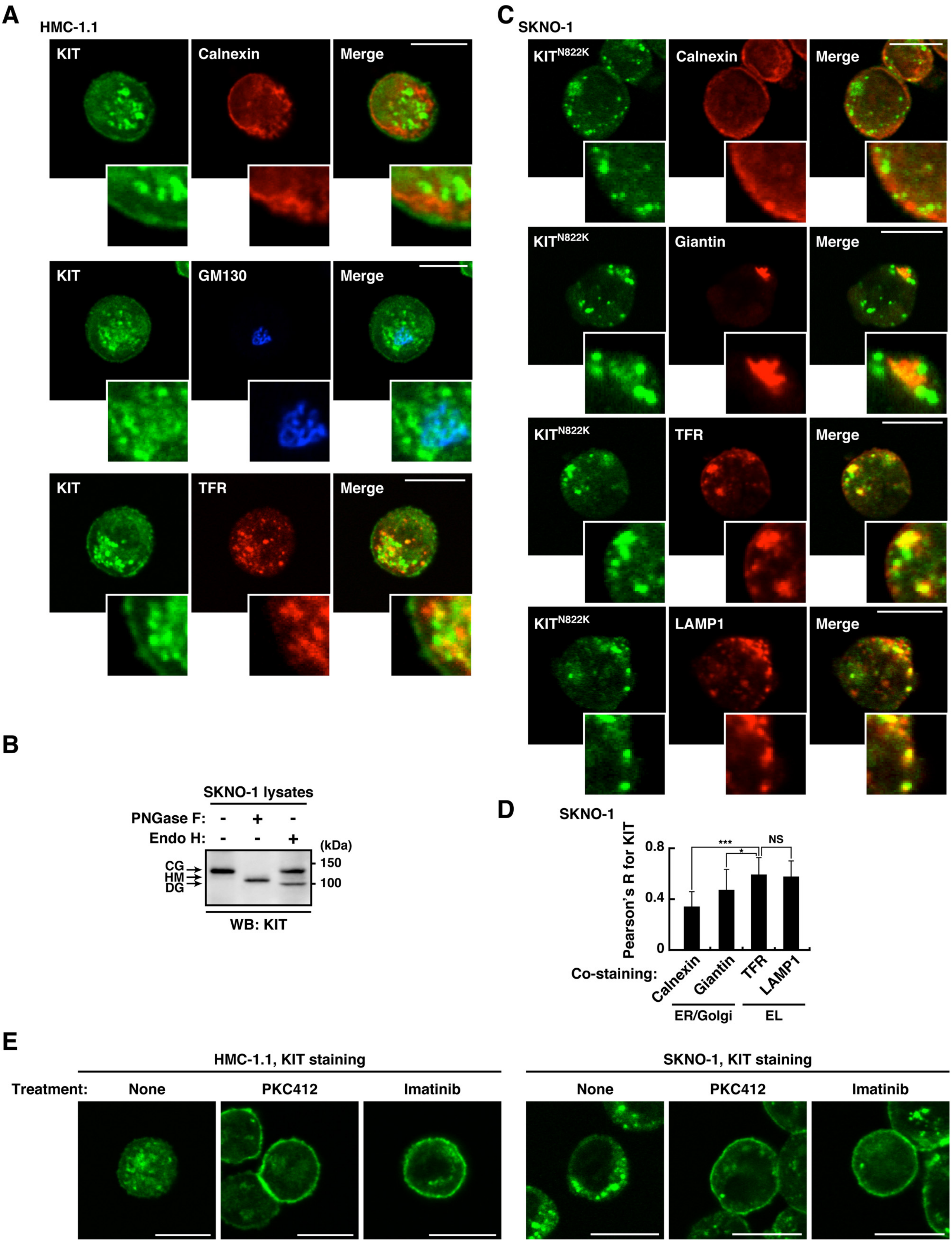

Figure S1. Mutant KIT localizes preferentially to EL in HMC-1.1 and SKNO-1 cells. **A** HMC-1.1 cells were double-stained with anti-KIT (green) plus the indicated antibody (red or blue). Insets show magnified images. Bars, 10 µm. Calnexin (ER marker); GM130 (Golgi marker), giantin (Golgi marker); TFR (endosome marker). **B** Lysates from SKNO-1 cells were treated with peptide N-glycosidase F (PNGase F) or endoglycosidase H (endo H) then immunoblotted with anti-KIT. CG, complex-glycosylated form; HM, high mannose form; DG, deglycosylated form. **C** SKNO-1 cells were double-stained with anti-KIT (green) plus the indicated antibody (red). Insets show magnified images. Bars, 10 µm. **D** Pearson's R correlation coefficients were calculated by analyzing the intensity of KIT *vs.* organelle markers. Results are means ± s.d. (n = 13~26). **P* < 0.05, ****P* < 0.001. NS, not significant. Calnexin (ER marker); giantin (Golgi marker); TFR (endosome marker); LAMP1 (lysosome marker). **E** HMC-1.1 (left panels) or SKNO-1 cells (right panels) were treated for 12 h with 1 µM PKC412 or 1 µM imatinib, then immunostained with anti-KIT. Bars, 10 µm.

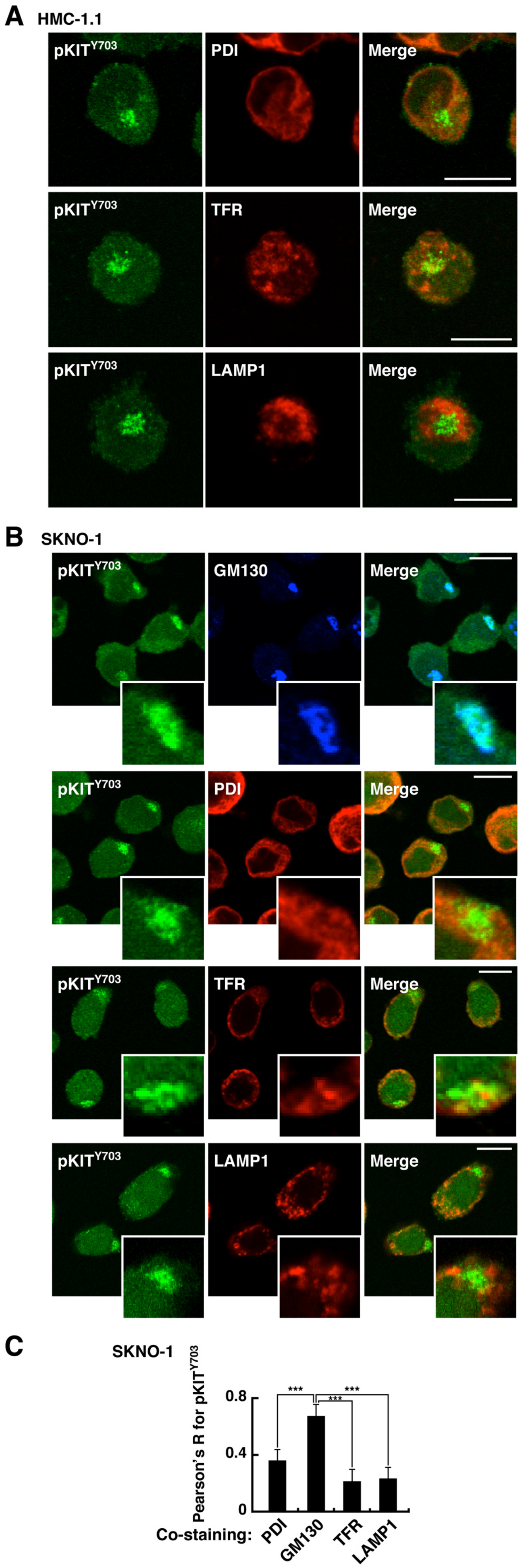

Figure S2. In HMC-1.1 and SKNO-1, the major site for KIT autophosphorylation is colocalized with the Golgi region. **A** & **B** HMC-1.1 (**A**) or SKNO-1 (**B**) cells were immunostained for phospho-KIT Y703 ($pKIT^{Y703}$, green) together with the indicated antibody (red or blue). GM130 (Golgi marker, blue), PDI (protein disulfide isomerase, ER marker, red), TFR (endosome marker, red), or LAMP1 (lysosome marker, red). Bars, 10 μm. **C** Pearson's R correlation coefficients were calculated by analyzing the intensity of $pKIT^{Y703}$ *vs*. organelle markers. Results are means ± s.d. (n = 15~19). ***$P < 0.001$.

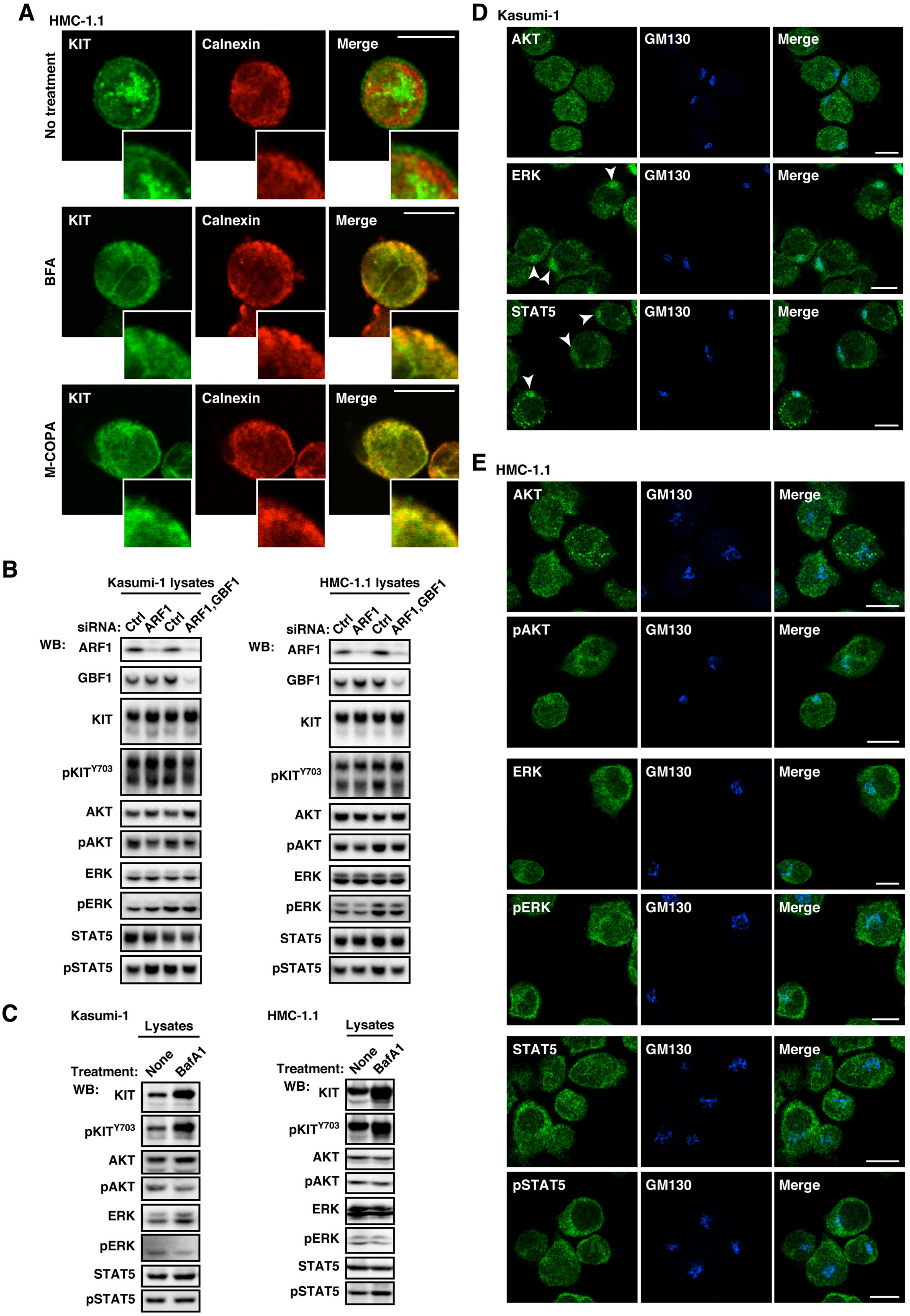

Figure S3. Distribution of signal molecules in Kasumi-1 and HMC-1.1 cells. **A** HMC-1.1 cells were treated for 16 h with 5 µM BFA or 1 µM M-COPA (inhibitors of ER export to the Golgi) then immunostained for KIT (green) and calnexin (ER marker, red). Bars, 10 µm. **B** Kasumi-1 (left) or HMC-1.1 (right) were transfected with siRNAs for 48 h to knock down *ARF1* and *GBF1*. Lysates were immunoblotted. **C** Kasumi-1 (left) or HMC-1.1 cells (right) were treated with 100 nM bafilomycin A1 (BafA1, an inhibitor of endosome-to-lysosome trafficking) for 12 h (Kasumi-1) or 24 h (HMC-1.1), and then immunoblotted. **D** & **E** Kasumi-1 (**D**) or HMC-1.1 (**E**) cells were immunostained with the indicated antibody. Arrowheads indicate the Golgi region. Bars, 10 µm.

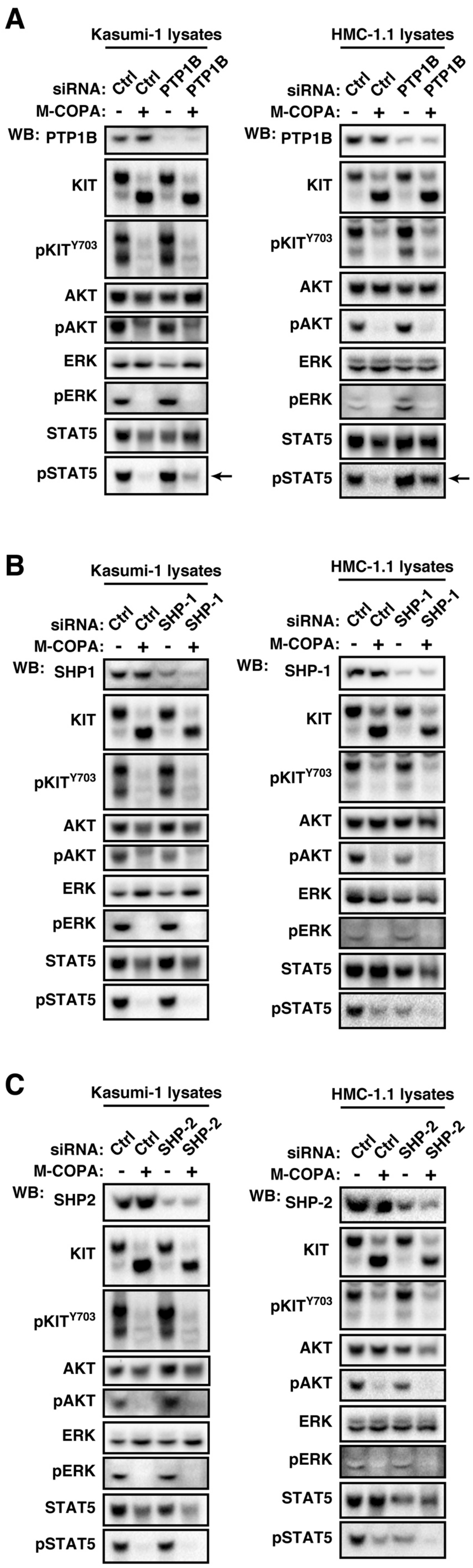

Figure S4. Effect of knockdown of *PTP1B*, *SHP-1*, and *SHP-2* on KIT signals. **A**-**C** Cells were transfected with *PTP1B*, *SHP-1*, or *SHP-2* siRNAs for 48 h and treated with 1 μM M-COPA (inhibitor of ER export) during the last 8 h (Kasumi-1) or 12 h (HMC-1.1). Lysates were immunoblotted.

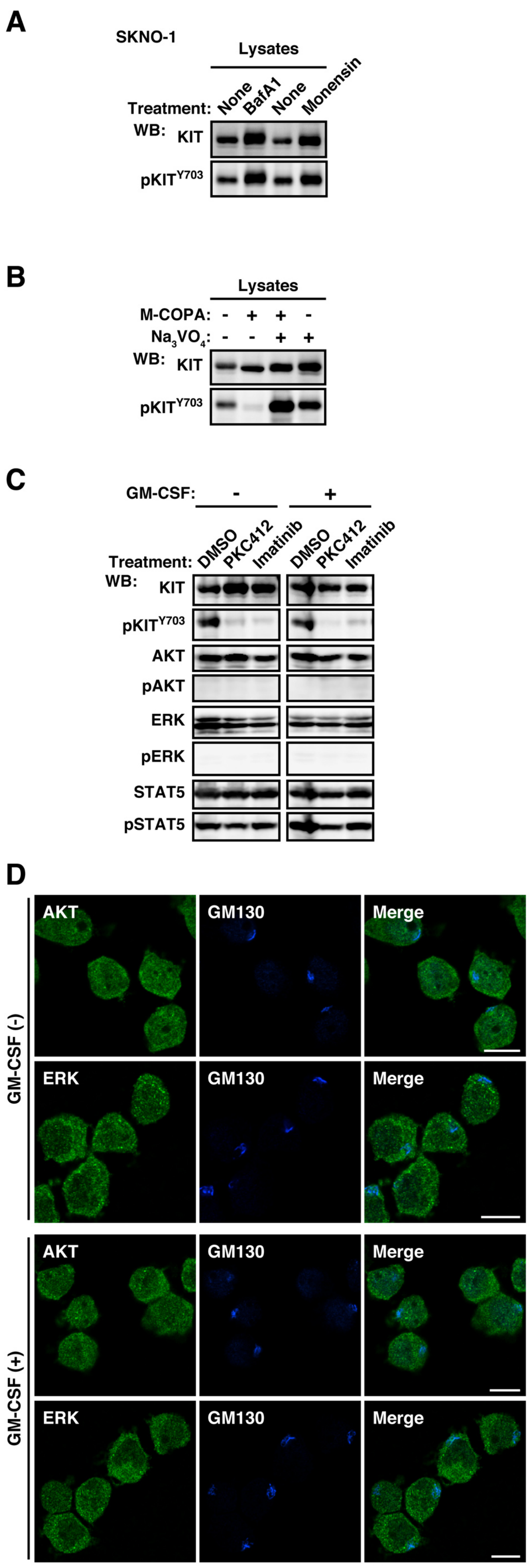

Figure S5. KIT^{N822K} does not activate AKT, ERK, and STAT5 in SKNO-1 cells. **A** SKNO-1 cells were treated with 100 nM bafilomycin A1 (BafA1, an inhibitor of endosome-to-lysosome trafficking) or 250 nM monensin (an inhibitor of *intra*-Golgi transport) for 12 h, and were then immunoblotted. **B** SKNO-1 cells were treated with 1 µM M-COPA (an inhibitor of ER export) for 12 h, and also with 3 mM Na_3VO_4 (an inhibitor of PTPs) during the last 3 h, and were then immunoblotted. **C** SKNO-1 cells were treated with 1 µM PKC412 or 1 µM imatinib for 4 h in the presence or absence of 10 ng/mL GM-CSF. Lysates were immunoblotted. **D** SKNO-1 cells cultured in the presence or absence of 10 ng/mL GM-CSF were immunostained with the indicated antibody. Bars, 10 µm.

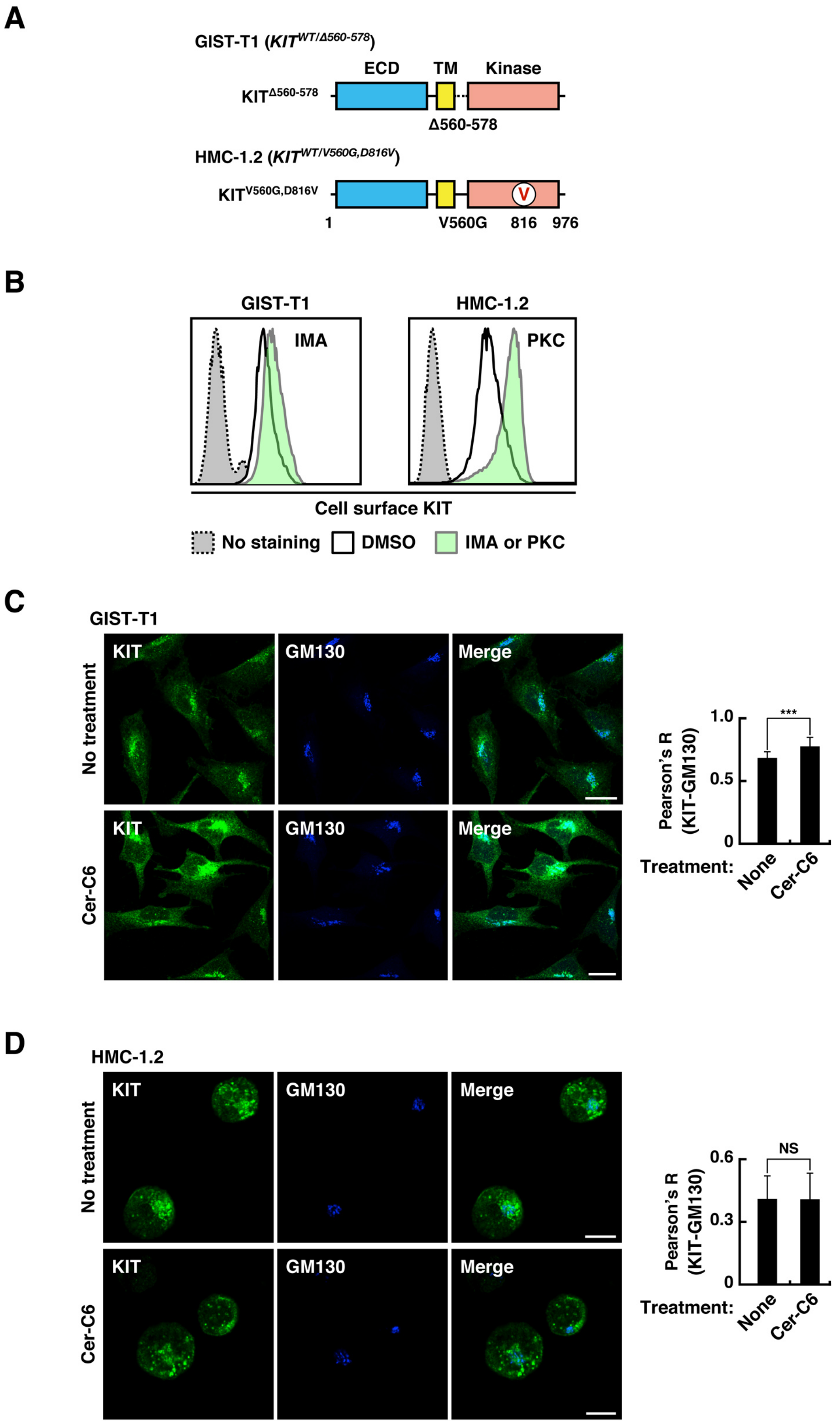

Figure S6. Effect of inhibition of normal lipid raft formation on KIT distribution. **A** Schematic representations of constitutively active KIT mutants ($KIT^{\Delta560\text{-}578}$ and $KIT^{V560G/D816V}$) showing the extracellular domain (ECD), the transmembrane domain (TM), the kinase domain, the deletion of 560-578 (dashed line), V560G, and D816V. **B** Cells were treated with the indicated TKIs (GIST-T1 together with 0.2 µM imatinib (IMA) for 4 h; HMC-1.2 with 1 µM PKC412 (PKC) for 12 h). Non-permeabilized cells were stained with anti-KIT extracellular domain antibody. Cell surface KIT levels determined by flow cytometry are shown. Green histogram, with KIT inhibitor treatment; white histogram, no KIT inhibitor; gray histogram, no anti-KIT antibody control. **C** GIST-T1 cells were treated with 0~10 µM cer-C6 for 10 h, and then immunostained for KIT (green) and GM130 (Golgi marker, blue). Bars, 20 µm. The graphs show Pearson's R (KIT-GM130). Results are means ± s.d. (n = 14~15). **P < 0.05, ***P < 0.001. **D** HMC-1.2 cells were treated with 0~40 µM cer-C6 for 8 h, then immunostained. Bars, 10 µm. The graphs show Pearson's R (KIT-GM130). Results are means ± s.d. (n = 18~23). NS, not significant.
